# Serological surveillance of parasitic zoonoses in 13 highlands regions of Peru: Period 2016-2019

**DOI:** 10.17843/rpmesp.2023.402.12472

**Published:** 2023-06-30

**Authors:** Isidro Antitupa, Nury Jakeline Vargas-Mayuri, Jhon Vicent Mayo, Luis Arturo Estares-Porras, William Marcelino Quispe Paredes, Elizabeth Luz Sánchez, Gilmer Solis-Sánchez

**Affiliations:** 1 National Referral Laboratory for Metaxenics and Parasitic Zoonoses, Instituto Nacional de Salud, Lima, Perú. National Referral Laboratory for Metaxenics and Parasitic Zoonoses Instituto Nacional de Salud Lima PerU; 2 Directorate of Prevention and Control of Metaxenic Diseases and Zoonoses of the General Directorate of Strategic Interventions in Public Health, Ministerio de Salud, Lima, Peru. Directorate of Prevention and Control of Metaxenic Diseases and Zoonoses of the General Directorate of Strategic Interventions in Public Health Ministerio de Salud Lima Peru; 3 Universidad Científica del Sur, Lima, Peru. Universidad Científica del Sur Universidad Científica del Sur Lima Peru

**Keywords:** Taenia solium Cysticercosis, Cystic Echinococcosis, Fascioliasis, ELISA, Seroepidemiologic Studies, Public Health Surveillance, Zoonoses, Peru

## Abstract

**Objectives.:**

To determine seropositivity to anti-IgG antibodies against Echinococcus granulosus, Fasciola hepatica and Taenia solium cysticercus infection and to describe the characteristics of the infected patients in 13 regions of the Peruvian highlands between 2016 and 2019.

**Materials and methods.:**

Cross-sectional, observational study, in which we analyzed 7811 epidemiological records of laboratory-based surveillance of parasitic zoonoses from 2016 to 2019. Diagnosis was established by detecting IgG type anti-E. granulosus, F. hepatica and T. solium cysticercus antibodies using native antigens by enzyme-linked immunosorbent assay (ELISA) and Immunoblot. We evaluated the difference in the frequency of the cases according to identified characteristics using Pearson’s chi-square test and Fisher’s exact test.

**Results.:**

Seropositivity was 7.9% for fascioliasis, 4.9% for cystic echinococcosis, and 2.3% for T. solium cysticercus. These rates were higher in Cerro de Pasco for cystic echinococcosis (24.5%), in Ayacucho for T. solium cysticercus (4.5%) and in Puno for fascioliasis (40.6%). Regarding the sociodemographic characteristics, we found a statistically significant difference in the frequency of cases for all zoonoses according to age group, occupation, and region of residence. We also found a difference with the consumption of vegetables in emollients, and between clinical-epidemiological characteristics and having a family history of parasitic zoonoses.

**Conclusions.:**

From the 7811 samples, we found that these parasitic zoonoses are distributed in 13 regions of the Peruvian highlands, and represent a major health problem, with frequencies that change according to different characteristics.

## INTRODUCTION

Parasitic zoonoses are neglected diseases that are naturally transmitted from vertebrate animals to humans, the most important being zoonoses caused by helminths such as *Fasciola hepatica*, *Echinococcus granulosus* and *Taenia solium*^1^. In humans, *T. solium cysticercus* infection mainly affects the central nervous system (CNS), *F*. *hepatica* infection affects the bile ducts of the liver, and *E. granulosus* infection affects the liver and lungs. All these tissue parasitosis are asymptomatic until the chronic phase of the disease, where the main signs and symptoms begin to manifest, significantly deteriorating health ^2^.

These zoonoses are mainly distributed in Africa, Asia, Southern Europe and South America ^(^^3^^-^^5^. In Peru, seroprevalence rates in humans of up to 20% of cystic echinococcosis were reported in Cerro de Pasco ^6^, 24% of *T. solium cysticercus* in Saylla (Cusco) ^7^ and up to 31% of fascioliasis in Puno ^8^, which are rural and cattle-raising areas of the central and southern highlands. It is estimated that, in Peru, the burden of disease per disability-adjusted life years (DALY) for human cystic echinococcosis is 1139 years with a total annual cost of 2,420,348 US dollars ^9^; however, there are no DALY estimates for *T. solium cysticercus* and human fascioliasis.

The main risk factors associated with human cystic echinococcosis, which have been reported in the Peruvian population, are exposure to infected dogs, contact with infected cattle and consumption of food contaminated with eggs of *E. granulosus*, which is the infecting form of the parasite ^10^. Human fascioliasis is associated with determinants such as exposure to infected cattle and consumption of raw vegetables exposed to irrigation water contaminated with metacercariae of *F. hepatica*, which is the infecting form of the trematode ^11^. On the other hand, the risk factors associated with *T. solium cysticercus* are the consumption of raw or undercooked pork contaminated with T*. solium cysticercus* larvae, consumption of food and water contaminated with feces of people with taeniasis through direct transmission of *T. solium* eggs, which is the infecting form of *T. solium cysticercus*^12^. In addition, there are common determinants for the three zoonoses such as deficient hygienic habits and unfavorable socioeconomic conditions, such as the lack of access to adequate health services, education and basic sanitation ^10^^-^^12^.

There are studies on the distribution of zoonoses at the national level focused on school-age children; however, neither the communities at high risk of zoonoses in the Peruvian territory nor the magnitude of the disease in the general population have been determined. For this reason, this study aimed to determine seropositivity to anti-IgG antibodies for *E. granulosus*, *F. hepatica* and *T. solium cysticercus* infection and to describe the characteristics of those infected in 13 regions of the Peruvian highlands between 2016 and 2019.

**KEY MESSAGES**

Motivation for the study. To understand the characteristics and distribution of the main parasitic zoonoses in Peru and to generate data for decision making in surveillance, prevention and control.

Main findings. These parasitic zoonoses are distributed in areas of extreme poverty in the central and southern highlands of Peru. Fascioliasis seropositivity was found to be higher than for echinococcosis and cysticercosis. In addition, sociodemographic characteristics and lifestyle habits influence the transmission of these zoonoses.

Implications. An active search for these zoonoses should be carried out in other risk areas with similar epidemiological characteristics to determine the prevalence of each of these zoonoses and implement multisectoral prevention and control programs.

## MATERIALS AND METHODS

### Study design

We conducted an observational, quantitative, of cross-sectional study, in which we analyzed the epidemiological records of parasitic zoonoses and results obtained from the activities of laboratory-based surveillance for the identification of parasitic zoonoses, executed by the National Referral Laboratory of Parasitic Zoonoses of the National Institute of Health (LRNZOP-INS) between the years 2016 and 2019.

### Study population

Epidemiological files of villagers who participated in laboratory-based epidemiological surveillance activities between 2016 and 2019 were evaluated. We included participants from 13 highland regions of Peru (Apurímac, Arequipa, Ayacucho, Cajamarca, Cerro de Pasco, Cusco, Huancavelica, Huánuco, Junín, Lima Provincias, Moquegua, Puno and Tacna), which are located between 1800 and 4100 meters above sea level, with an estimated total poverty rate between 23 and 44% ^13^, and are characterized by having livestock and agriculture as their main economic activity.

### Sample and selection of participants

The analysis included all the available epidemiological files from each region. The assessment of the files was based on the number of inhabitants in each locality and the logistical capacity of the Regional Health Directorates (DIRESA) of the regions that implemented the surveillance activity. Screening was carried out by convenience by proactive search of participants in homes and educational institutions in each locality. The screening considered people older than five years of age and with permanent residence in endemic areas, while people with other conditions diagnosed by laboratory tests were excluded. We excluded incomplete files.

### Data collection

We used data that had been previously collected for the epidemiological surveillance of parasitic zoonoses: cystic echinococcosis, fascioliasis and *T. solium cysticercus* (Supplementary Material), as well as the results of the serological tests of each of the screened participants registered in the NETLAB system.

The epidemiological files analyzed were prepared by LRNZOP-INS for surveillance activities. These files have been previously reviewed by technical experts, were applied by trained personnel, and were stored at LRNZOP-INS, as a tool for the elaboration of technical reports on the epidemiological situation of these parasitic diseases.

### Serological data

After filling out the epidemiological record, we obtained a 5 mL sample of venous blood for the serological diagnosis by ELISA-IgG and Immunoblot-IgG. It should be noted that both diagnostic kits were produced at LRNZOP-INS. The Immunoblot method for cystic echinococcosis has a sensitivity of 95% and specificity of 100% for the 8 kDa, 16 kDa, and 21 kDa bands, using antigens from the hydatid fluid of *E. granulosus*. On the other hand, the immunoblot for *T. solium* cysticercus has a sensitivity of 93% and specificity of 100% for the bands of 13 kDa, 14 kDa, 17 kDa, 18 kDa, 23 kDa, 24 kDa, 31 kDa, and 35 kDa, using antigens from the vesicular fluid of *T. solium* cysticercus. Finally, the immunoblot for fascioliasis has a sensitivity of 91% and specificity of 99%, using purified antigens of 27-28 kDa from the secretion/excretion products of F. hepatica ^14^^,^^15^.

First, serological screening was performed for each parasite, using the ELISA-IgG method for cystic echinococcosis, fascioliasis and *T. solium* cysticercus, which were carried out in the Regional Reference Laboratories (LRR) of each DIRESA. Then, diagnosis was confirmed by using the Immunoblot-IgG method for cystic echinococcosis, fascioliasis, and *T. solium* cysticercus of the samples with REACTIVE results in the ELISA-IgG method, which were performed at the LRNZOP-INS.

### Variables

The dependent variable corresponds to the categorical result (positive, negative) of the serological diagnosis of zoonoses according to the type of parasitosis. The independent variables correspond to data obtained from epidemiological files that have been of interest to other studies for this type of infections ^11^^,^^16^^-^^20^. These variables included information on sociodemographic characteristics, animal husbandry, slaughtering and risk practices, consumption of risky foods and beverages, and clinical and epidemiological characteristics.

The names of some of the variables were adapted from the names of the items on the files for an adequate presentation. Some original items incorporated the alternative “other” to allow an exhaustive collection of responses that were considered, at the discretion of the LRNZOP-INS team, to be infrequent at the time of the file’s elaboration. Among these variables are “Housing material” (which could include stone with mud, cardboard, stone or ashlar, etc.), as well as “Type of vegetables consumed” and “Type of vegetable consumption”. The characteristics of the variable adjustments/groupings are presented in the Supplementary Material.

We assessed the following sociodemographic characteristics: age, sex, occupation, educational level, housing material, source of water for human consumption, consumption of boiled water, type of toilet facilities, and department of origin, as well as characteristics related to animal husbandry, slaughtering and risk practices. Data were obtained on the type of animals raised currently or previously, type of raising and place of slaughter of pigs, place of slaughter of sheep/bovine/goats, feeding raw offal to their dogs, deworming of their dogs and handling of meat with cysticercosis. Age was categorized according to life stage, which is a classification widely used in health systems. Occupation was categorized by convenience considering the similarity and affinity of answers found in the epidemiological file.

Information on the foods consumed, the frequency of pork and vegetable consumption, type of vegetables consumed and form of consumption was also collected. The clinical and epidemiological characteristics evaluated included personal semiological history (fainting, weight loss, abdominal pain, headache, chest pain, epilepsy, fever, jaundice, dizziness, nausea, chronic cough and vomiting), and family history of echinococcosis, cysticercosis and fascioliasis.

### Statistical analysis

Data analysis was carried out with the Stata v17.0 statistical package (Stata Corporation, College Station, Texas, USA). Descriptive estimates of the study variables were made by obtaining absolute and relative frequencies. In an exploratory manner, the difference in the proportion of each infection was identified according to all the study characteristics by means of Pearson’s chi-square test or Fisher’s Exact test (according to Cochran’s rule). These tests were performed considering a significance level of 0.05.

### Ethical Aspects

The present study used secondary data that were collected during epidemiological surveillance activities in the framework of the “Protocol for Laboratory-Based Surveillance of Parasitic Zoonoses (Teniosis/Cysticercosis, Cystic Echinococcosis, and Fasciolosis)” approved in 2015 by the National Public Health Center of the INS.

Prior to obtaining clinical and laboratory information, as established in the aforementioned protocol, each participant gave their authorization through consent and/or informed assent.

## RESULTS

**Figure 1:**
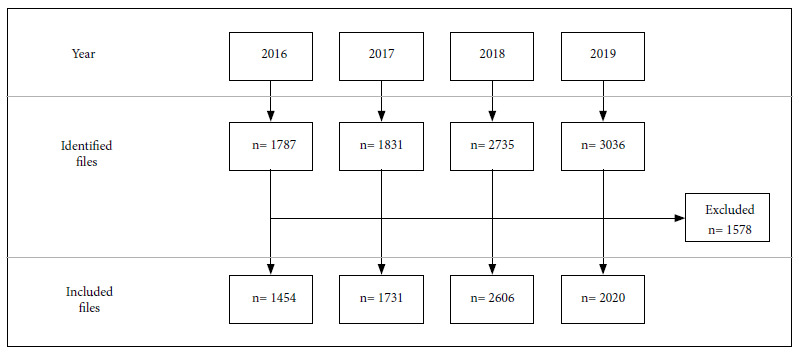
Flow chart of the analyzed epidemiological files.

Of the 9389 epidemiological files identified, 1578 were excluded due to lack of information. A total of 7811 epidemiological files obtained from inhabitants of 13 highland regions of Peru were included (Figure 1).

Table 1 describes the sociodemographic characteristics of the population; 36.7% were adults, 65% were male, and 41.4% were students. Likewise, most the participants had primary education (44.4%), had adobe as a housing wall material (85.2%), had water supply through a sink or pipe (52.3%), had access to boiled water (86.1%), a bathroom with a drain (50.5%) and were from Apurimac (26.2%).

**Table 1:** Sociodemographic characteristics of the studied population from 13 highland regions of Peru, 2016-2019.

Sociodemographic characteristics	Total number of evaluated files		Cystic echinococcosis positive	p-value	Cysticercosis positive	p-value	Fascioliasis positive	p-value
	n / N	%	n/N (%)		n/N (%)		n/N (%)	
Age group								
Children (5 -11 years)	1241 / 7811	15.9	42 / 1241 (3.4)	0.031^a^	10 / 1241 (0.8)	<0.001^a^	96 / 1241 (7.7)	<0.001^a^
Adolescents (12 -17 years)	1774 / 7811	22.7	77 / 1774 (4.3)		25 / 1774 (1.4)		172 / 1774 (9.7)	
Young adults (18 - 29 years)	974 / 7811	12.5	53 / 974 (5.4)		24 / 974 (2.5)		76 / 974 (7.8)	
Adults (30 - 59 years)	2866 / 7811	36.7	157 / 2866 (5.5)		83 / 2866 (2.9)		178 / 2866 (6.2)	
Older adults (60 or more)	956 / 7811	12.2	52 / 956 (5.4)		39 / 956 (4.1)		93 / 956 (9.7)	
Sex								
Women	2730 / 7811	35.0	129 / 2730 (4.7)	0.647^a^	51 / 2730 (1.9)	0.053^a^	214 / 2730 (7.8)	0.934^a^
Men	5081 / 7811	65.0	252 / 5081 (5.0)		130 / 5081 (2.6)		401 / 5081 (7.9)	
Occupation								
Student	3230 / 7811	41.4	136 / 3230 (4.2)	0.019^a^	37 / 3230 (1.1)	<0.001^a^	280 / 3230 (8.7)	<0.001^a^
Trade	671 / 7811	8.6	23 / 671 (3.4)		20 / 671 (3.0)		22 / 671 (3.3)	
Housekeeping/cleaning	1635 / 7811	20.9	93 / 1635 (5.7)		67 / 1635 (4.1)		94 / 1635 (5.7)	
Agriculture and Livestock	2248 / 7811	28.8	128 / 2248 (5.7)		56 / 2248 (2.5)		219 / 2248 (9.7)	
Not registered	27 / 7811	0.3	1 / 27 (3.7)		1 / 27 (3.7)		0 / 27 (0.0)	
Education level								
Primary	3278 / 7389	44.4	149 / 3278 (4.5)	0.554^a^	81 / 3278 (2.5)	0.194^a^	262 / 3278 (8.0)	0.034^a^
Secondary	3242 / 7389	43.9	159 / 3242 (4.9)		68 / 3242 (2.1)		258 / 3242 (8.0)	
Higher	604 / 7389	8.2	28 / 604 (4.6)		10 / 604 (1.7)		28 / 604 (4.6)	
No education	265 / 7389	3.6	17 / 265 (6.4)		10 / 265 (3.8)		21 / 265 (7.9)	
Housing material								
Adobe	6144 / 7210	85.2	272 / 6144 (4.4)	0.519^a^	152 / 6144 (2.5)	0.064^a^	502 / 6144 (8.2)	<0.001^a^
Hut	126 / 7210	1.7	9 / 126 (7.1)		5 / 126 (4.0)		14 / 126 (11.1)	
Noble materials	802 / 7210	11.1	35 / 802 (4.4)		9 / 802 (1.1)		31 / 802 (3.9)	
Other	138 / 7210	1.9	7 / 138 (5.1)		4 / 138 (2.9)		5 / 138 (3.6)	
Source of water for human consumption								
Drinking water	2427 / 6486	37.4	117 / 2427 (4.8)	0.200^a^	65 / 2427 (2.7)	0.304^a^	196 / 2427 (8.1)	<0.001^a^
Fountain and/or pipe	3393 / 6486	52.3	145 / 3393 (4.3)		77 / 3393 (2.3)		230 / 3393 (6.8)	
Well	301 / 6486	4.6	10 / 301 (3.3)		9 / 301 (3.0)		37 / 301 (12.3)	
Spring	245 / 6486	3.8	11 / 245 (4.5)		8 / 245 (3.3)		22 / 245 (9.0)	
Irrigation canal	49 / 6486	0.8	5 / 49 (10.2)		0 / 49 (0.0)		6 / 49 (12.2)	
River	71 / 6486	1.1	1 / 71 (1.4)		4 / 71 (5.6)		0 / 0 (0.0)	
Consumes boiled water								
Yes	6212 / 7213	86.1	305 / 6212 (4.9)	0.027†	146 / 6212 (2.4)	0.857^a^	457 / 6212 (7.4)	0.024†
No	855 / 7213	11.9	33 / 855 (3.9)		18 / 855 (2.1)		72 / 855 (8.4)	
Sometimes boiled or unboiled	146 / 7213	2.0	1 / 146 (0.7)		4 / 146 (2.7)		19 / 146 (13.0)	
Bathroom								
Toilet with drainage	3592 / 7111	50.5	169 / 3592 (4.7)	0.445^a^	79 / 3592 (2.2)	0.353^a^	207 / 3592 (5.8)	<0.001^a^
Latrine	2987 / 7111	42.0	123 / 2987 (4.1)		73 / 2987 (2.4)		262 / 2987 (8.8)	
Defecate in open field	532 / 7111	7.5	21 / 532 (3.9)		17 / 532 (3.2)		70 / 532 (13.2)	
Department of origin								
Lima Provinces	1187 / 7811	15.2	73 / 1187 (6.1)	<0.001^a^	16 / 1187 (1.3)	<0,001^a^	155 / 1187 (13.1)	<0.001^a^
Huánuco	903 / 7811	11.6	17 / 903 (1.9)		17 / 903 (1.9)		78 / 903 (8.6)	
Junín	528 / 7811	6.8	28 / 528 (5.3)		9 / 528 (1.7)		29 / 528 (5.5)	
Apurímac	2047 / 7811	26.2	67 / 2047 (3.3)		36 / 2047 (1.8)		121 / 2047 (5.9)	
Cusco	1318 / 7811	16.9	57 / 1318 (4.3)		50 / 1318 (3.8)		91 / 1318 (6.9)	
Ayacucho	969 / 7811	12.4	78 / 969 (8.0)		44 / 969 (4.5)		60 / 969 (6.2)	
Huancavelica	197 / 7811	2.5	14 / 197 (7.1)		1 / 197 (0.5)		7 / 197 (3.6)	
Cajamarca	140 / 7811	1.8	0 / 140 (0.0)		0 / 140 (0.0)		28 / 140 (20.0)	
Arequipa	188 / 7811	2.4	7 / 188 (3.7)		2 / 188 (1.1)		29 / 188 (15.4)	
Moquegua	14 / 7811	0.2	0 / 14 (0.0)		0 / 14 (0.0)		0 / 14 (0.0)	
Tacna	133 / 7811	1.7	1 / 133 (0.8)		0 / 133 (0.0)		2 / 133 (1.5)	
Puno	32 / 7811	0.4	1 / 32 (3.1)		0 / 32 (0.0)		13 / 32 (40.6)	
Cerro de Pasco	155 / 7811	2.0	38 / 155 (24.5)		6 / 155 (3.9)		2 / 155 (1.3)	
Total	----	----	381 / 7811 (4.9)		181 / 7811 (2.3)		615 / 7811 (7.9)	

a Pearson’s Chi-square test.

The frequency of cases of cystic echinococcosis, *T. solium* cysticercus infection and fascioliasis was 4.9%, 2.3% and 7.9%, respectively. In addition, the highest number of cases of cystic echinococcosis was found in adults (5.5%), most cases of *T. solium* cysticercus infection were found in older adults (4.1%), and most cases of fascioliasis were reported in adolescents and older adults (9.7%). The occupational groups most affected by cystic echinococcosis were housewives and farmers (5.7%), housewives had the most cases of *T. solium* cysticercus infection (4.1%), and farmers had the most cases of fascioliasis (9.7%). Significant positivity for fascioliasis was found predominantly in people with primary and secondary education (8.0%). Likewise, the highest frequency of fascioliasis was found in participants residing in hut dwellings (11.1%), as well as in those who received accessed water by wells (12.3%), and defecated in the open field (13.2%). The highest frequency of cystic echinococcosis was found in Cerro de Pasco (24.5%), of cysticercosis in Ayacucho (4.5%), and of fascioliasis in Puno (40.6%) (Table 1).

In terms of people’s habits and activities, significant positivity for cystic echinococcosis was found in participants who raised pigs (5.4%) and sheep (3.8%); and for fascioliasis, in farmers who raised goats (7.2%), cows (6.2%) and sheep (6.5%). The highest frequency of cysticercosis was found in participants who raised free-range pigs (4.1%), and those who slaughtered their pigs near their homes (2.4%). People with did not deworm their dogs had a higher frequency of cystic echinococcosis (6.0%), while cysticercosis cases were higher in those who sold meat contaminated with *T. solium* cysticercus (5.7%) (Table 2).

**Table 2:** Distribution of parasitic zoonoses according to animal husbandry characteristics, slaughtering and risk practices in 13 highland regions of Peru, 2016-2019.

Animal husbandry, slaughtering and risk practices	Total number of evaluated files		Cystic echinococcosis positive n/N (%)	p-value	Cysticercosis positive n/N (%)	p-value	Fascioliasis positive n/N (%)	p-value
	n / N	%						
Animals raised or bred								
Pigs	4022 / 7202	55.8	217/4022 (5.4)	<0.001^a^	99 / 4022 (2.5)	0.415^a^	310 / 4022 (7.7)	0.649^a^
Goats	6447 / 7121	90.5	283/6447 (4.4)	0.543^a^	147 / 6447 (2.3)	0.877^a^	462 / 6447 (7.2)	<0.001^a^
Cows	3583 / 7194	49.8	164/3583 (4.6)	0.597^a^	77 / 3583 (2.1)	0.373^a^	221 / 3583 (6.2)	<0.001^a^
Sheep	4054 / 7209	56.2	153/4054 (3.8)	<0.001^a^	84 / 4054 (2.1)	0.163^a^	263 / 4054 (6.5)	<0.001^a^
Dogs	1793 / 7247	24.7	83/1793 (4.6)	0.787^a^	47 / 1793 (2.6)	0.326^a^	117 / 1793 (6.5)	0.069^a^
Type of pig breeding								
At home in a corral	1452 / 3423	42.4	82/1452 (2.7)	0.271^a^	27 / 1452 (1.9)	0.001^a^	117 / 1452 (8.1)	0.617^a^
In a field corral	1140 / 3423	33.3	43/1140 (3.8)		19 / 1140 (1.7)		92 / 1140 (8.1)	
In the open field	831 / 3423	24.3	29/831 (3.5)		34/831 (4.1)		76 / 831(9.1)	
Place of pig slaughter								
Peridomicile	2968 / 3263	91.0	96/2968 (3.2)	0.445^a^	71 / 2968 (2.4)	0.022^a^	249 / 2968 (8.4)	0.580^a^
Slaughterhouse	295 / 3263	9.0	12/295 (4.1)		1 / 295 (0.3)		22 / 295 (7.5)	
Sheep/cow/goat slaughtering place								
Peridomicile	3558 / 3840	92.7	149/3558 (4.2)	0.957†	97 / 3558 (2.7)	0.040†	319 / 3558 (9.0)	0.204†
Slaughterhouse	282 / 3840	7.3	12/282 (4.3)		2 / 282 (0.7)		19 / 282 (6.7)	
Feeds raw offal to the dogs								
No	2026 / 4879	41.5	104/2026 (5.1)	0.043†	49 / 2026 (2.4)	0.516†	188 / 2026 (9.3)	0.051†
Yes	2853 / 4879	58.5	112/2853 (3.9)		61 / 2853 (2.1)		220 / 2853 (7.7)	
Dewormed dog								
No	1451 / 4834	30.0	87/1451 (6.0)	<0.001^a^	39 / 1451 (2.7)	0.162^a^	103 / 1451 (7.1)	0.092^a^
Yes	3383 / 4834	70.0	112/3383 (3.3)		69 / 3383 (2.0)		289 / 3383 (8.5)	
Knowledge about the disease:								
Fascioliasis								
No	2715 / 6579	41.3	132/2715 (4.9)	0.027^a^	84/2715 (3.1)	0.001^a^	215/2715 (7.9)	0.939^a^
Yes	3864 / 6579	58.7	145/3864 (3.8)		72 / 3864 (1.9)		304 / 3864 (7.9)	
Echinococcosis								
No	1731 / 4959	34.9	83/1731 (4.8)	0.035^a^	58/1731 (3.4)	<0.001^a^	132/1731 (7.6)	0.082^a^
Yes	3228 / 4959	65.1	115/3228 (3.6)		56 / 3228 (1.7)		293/3228 (9.1)	
Cysticercosis								
No	2754 / 5794	47.5	134/2754 (4.9)	0.009^a^	80/2754 (2.9)	0.004^a^	208/2754 (7.6)	0.106^a^
Yes	3040 / 5794	52.5	106/3040 (3.5)		54/3040 (1.8)		265/3040 (8.7)	
What happens with the meat with cysticercosis								
Eats it	342 / 3238	10.6	10/342 (2.9)	0.397^a^	12 / 342 (3.5)	0.019^a^	23/342 (6.7)	0.872^a^
Sells it	123 / 3238	3.8	4/123 (3.3)		7 / 123 (5.7)		9/123 (7.3)	
Burries it	2430 / 3238	75.0	114/2430 (4.7)		61 / 2430 (2.5)		192/2430 (7.9)	
Feeds it to the dog	343 / 3238	10.6	13/343 (3.8)		3 / 343 (0.9)		25/343 (7.3)	

a Pearson’s Chi-square test.

Regarding consumption of foods and beverages, most cases of cysticercosis were found among participants who consumed pork (2.9%); however, this difference was not statistically significant. In addition, participants who consumed emollients and vegetable extracts had a higher frequency of fascioliasis and cystic echinococcosis (p<0.05). Additionally, we evidenced a difference in cases of cystic echinococcosis by consumption of vegetables in juices (p<0.05) (Table 3).

**Table 3:** Distribution of parasitic zoonoses according to consumption of food and beverages at risk for parasitic zoonoses found in 13 highland regions of Peru, 2016-2019.

Consumption of food and beverages at risk for parasitic zoonosis	Total number of evaluated files		Cystic echinococcosis positive	p-value	Cysticercosis positive	p-value	Fascioliasis positive	p-value
	n / n	%	n/N (%)		n/N (%)		n/N (%)	
Food-related risk factors								
Consumes pork meat	1329 / 4938	26.9	76/1329 (5.7)	0.006^a^	38 / 1329 (2.9)	0.295^a^	114 / 1329 (8.6)	0.173^a^
Consumes raw vegetables	568 / 4938	11.5	19/568 (3.3)	0.169^a^	14 / 568 (2.5)	0.815^a^	42 / 568 (7.4)	0.853^a^
Frequency of pork consumption								
One to two times a month	1849 / 5031	36.8	67/1849 (3.6)	0.199^a^	31 / 1849 (1.7)	0.092^a^	129 / 1849 (7.0)	0.169^a^
One to two times a year	3182 / 5031	63.2	139/3182 (4.4)		76 / 3182 (2.4)		256 / 3182 (8.0)	
Frequency of vegetable consumption								
Every day	906 / 5441	16.7	28/906 (3.1)	0.271^a^	15 / 906 (1.7)	0.397^a^	69 / 906 (7.6)	0.102^a^
One to two times a month	3076 / 5441	56.5	134/3076 (4.4)		79 / 3076 (2,6)		287 / 3076 (9.3)	
Three to four times a month	1356 / 5441	24.9	64/1356 (4.7)		29 / 1356 (2.1)		99 / 1356 (7.3)	
One to two times a year	103 / 5441	1.9	5/103 (4.9)		3 / 103 (2.9)		8 / 103 (7.8)	
Vegetables consumed								
Watercress	1949 / 5614	34.7	93/1949 (4.8)	0.399^a^	50 / 1949 (2.6)	0.401^a^	155 / 1949 (8.0)	0.708^a^
Lettuce	4275 / 5614	76.1	194/4275 (4.5)	0.582^a^	102 / 4275 (2.4)	0.641^a^	337 / 4275 (7.9)	0.208^a^
Dandelion	112 / 5614	2.0	7/112 (6.3)	0.348^b^	2 / 112 (1.8)	1.000^b^	7 / 112 (6.3)	0.460^a^
Alfalfa	162 / 5614	2.9	5/162 (3.1)	0.392^a^	1 / 162 (0.6)	0.186^b^	10 / 162 (6.2)	0.353^a^
Other	18 / 5614	0.3	1/18 (5.6)	0.560^b^	1 / 18 (5.6)	0.347^b^	0 / 18 (0.0)	0.393^b^
Vegetable consumption								
Salads	5231 / 5614	93.2	234/5231 (4.5)	0.786^a^	120 / 5231 (2.3)	0.469^a^	429 / 5231 (8.2)	0.538^a^
Juice	420 / 5614	7.5	36/420 (8.6)	<0.001^a^	10 / 420 (2.4)	0.947^a^	34 / 420 (8.1)	0.972^a^
Extracts	210 / 5614	3.7	17/210 (8.1)	0.009^a^	1 / 210 (0.5)	0.096^b^	4 / 210 (1.9)	0.001^a^
Emollients	235 / 5614	4.2	4/235 (1.7)	0.037^a^	1 / 235 (0.4)	0.048^a^	6 / 235 (2.6)	0.001^a^
Other	66 / 5614	1.2	3/66 (4.5)	0.769^b^	3 / 66 (4.5)	0.199^b^	4 / 66 (6.1)	0.534^a^

a Pearson’s chi-squared test.

b Fisher’s exact test.

The main symptoms for cystic echinococcosis were weight loss (4.3%), chest pain (4.2%), and abdominal pain (4.4%). For cysticercosis, the main symptoms were headache (1.8%), dizziness (2.0%), and nausea (2.2%), while for fascioliasis they were weight loss (7.6%), abdominal pain (8.0%), fever (7.9%), and jaundice (7.8%). Regarding the assessment of family history, a statistically significant difference was found in the frequency of cases (p<0.05) for the three parasitic zoonoses (Table 4).

**Table 4:** Clinical and epidemiological characteristics of parasitic zoonoses found in 13 highland regions of Peru, 2016-2019.

Clinical and epidemiological characteristics	Total number of evaluated files		Cystic echinococcosis positive	p-value	Cysticercosis positive	p-value	Fascioliasis positive	p-value
	n / n	%	n/N (%)		n/N (%)		n/N (%)	
Signs and Symptoms								
Weight loss	5540 / 6246	88.7	238/5540 (4.3)	0.005 ^a^	122 / 5540 (2.2)	0.047 ^a^	420 / 5540 (7.6)	0.010 ^a^
Abdominal pain	3320 / 6235	53.2	146/3320 (4.4)	0.374 ^a^	71 / 3320 (2.1)	0.224 ^a^	265 / 3320 (8.0)	0.792 ^a^
Headache	3306 / 6220	53.2	133/3306 (4.0)	0.018 ^a^	59 / 3306 (1.8)	0.001 ^a^	262 / 3306 (7.9)	0.997 ^a^
Chest pain	5013 / 6184	81.1	213/5013 (4.2)	0.008 ^a^	111 / 5013 (2.2)	0.161 ^a^	409 / 5013 (8.2)	0.223 ^a^
Epilepsy	6189 / 6260	98.9	272/6189 (4.4)	0.770 ^b^	139 / 6189 (2.2)	0.675 ^b^	386 / 6189 (6.2)	<0.001 ^b^
Fever	4858 / 6247	77.8	218/4858 (4.5)	0.521 ^a^	108 / 4858 (2.2)	0.265 ^a^	384 / 4858 (7.9)	0.986 ^a^
Jaundice	6049 / 6241	96.9	277/6049 (4.6)	0.787 ^a^	137 / 6049 (2.3)	0.088 ^b^	471 / 6049 (7.8)	0.063 ^a^
Dizziness	4968 / 6252	79.5	228/4968 (4.6)	0.912 ^a^	98 / 4968 (2.0)	<0.001 ^a^	382 / 4968 (7.7)	0.298 ^a^
Nausea	4883 / 6200	78.8	215/4883 (4.4)	0.129 ^a^	105 / 4883 (2.2)	0.028 ^a^	377 / 4883 (7.7)	0.228 ^a^
Chronic cough	5417 / 6238	86.8	237/5417 (4.4)	0.060 ^a^	122 / 5417 (2.3)	0.330 ^a^	411 / 5417 (7.6)	0.024 ^a^
Vomiting	5282 / 6247	84.6	244/5282 (4.6)	0.612 ^a^	120 / 5282 (2.3)	0.322 ^a^	399 / 5282 (7.6)	0.027 ^a^
Family history								
Echinococcosis	6289 / 6548	96.0	272/6289 (4.3)	0.001 ^a^	147 / 6289 (2.3)	0.436 ^a^	503 / 6289 (8.0)	0.872 ^a^
Cysticercosis	6100 / 6526	93.5	274/6100 (4.5)	0.976 ^a^	133 / 6100 (2.2)	0.001 ^a^	505 / 6100 (8.3)	0.003 ^a^
Fascioliasis	6574 / 6658	98.7	287/6574 (4.4)	<0.001 ^a^	150 / 6574 (2.3)	0.711 ^a^	504 / 6574 (7.7)	<0.001 ^a^

a Pearson’s Chi-Square test.

b Fisher’s Exact Test.

## DISCUSSION

The National Referral Laboratory for Parasitic Zoonoses of the National Institute of Health decided to conduct laboratory-based surveillance to determine the extent of infection of fascioliasis, cystic echinococcosis, and *T. solium* cysticercosis in 7811 samples from 13 regions of Peru. Data from this activity have identified a seropositivity of 4.9% for cystic echinococcosis, 7.9% for human fascioliasis, and 2.3% for *T. solium* cysticercosis.

Cerro de Pasco (24.5%), Ayacucho (8%), Huancavelica (7.1%), Provinces of Lima (6.1%), Junín (5.3%) and Cusco (4.3%) had the highest cystic echinococcosis seropositivity, this maybe be due to the fact that they are considered endemic areas, as evidenced by other studies conducted in the Peruvian Andes ^21^^-^^24^. In addition, cases of cystic echinococcosis were found in Huánuco (1.9%), Tacna (0.8%), Arequipa (3.7%), Apurímac (3.3%) and Puno (3.1%). It is worth mentioning that we only obtained a small number of samples from the regions of Puno and Moquegua because these regions did not have adequate logistics to obtain a larger number of samples for laboratory-based surveillance of parasitic zoonoses.

The regions of Huánuco (8.6%), Ayacucho (6.2%) and Apurímac (5.9%) had the highest frequency of cases of human fascioliasis; however, we should also highlight the presence of cases in Cerro de Pasco (1.3%), Huancavelica (3.6%) and Tacna (1.5%), which is important because our study is the first report of cases of human fascioliasis in these regions. Therefore, it is of utmost importance to carry out further studies to identify risk areas for the control and prevention of this zoonosis.

The frequency of human fascioliasis was higher in Puno (40.6%), Cajamarca (20.0%), Arequipa (15.4%), Junín (5.5%) and Cusco (6.9%), which have been identified as endemic for this infection, as evidenced by Marcos *et al*. in their report of human cases between 1995 and 2005 ^25^, and by other studies that identified these areas as endemic ^16^^,^^26^^,^^27^. In addition to the aforementioned regions, we also found high seropositivity (13.1%) in Lima provinces. This finding is consistent with previous studies; a prevalence of 12.1% has been previously reported in Vichaycocha, Huancapón and Cajamarquilla, in the highlands of Lima ^28^. Likewise, the presence of this parasite has also been reported from coprological samples obtained in Huarochirí (16.7%) ^29^ and Oyón (1.12%) ^30^.

We found a 2.3% seropositivity rate of *T. solium* cysticercosis, which is lower than the estimated prevalence in Latin America (4.08%) ^3^. In addition, we identified cases of this infection in regions where it had not been previously reported, such as Huánuco (1.9%) and Huancavelica (0.5%). A seropositivity rate lower than pre-existing reports was evidenced in some regions, such as Cerro de Pasco which previously reported 4.2%, Cusco with 24.0% in Saylla, Junín with 7.0% in Huancayo, Apurímac with 12.0% in Andahuaylas, Tacna with 1.85% and Puno with 1.64% ^7^. On the other hand, there were regions with higher seropositivity rates than previously reported, such as Ayacucho, which had reports of 3.3% in Pampa Cangallo, Lima with previous seropositivity of less than 1%, and Arequipa, which previously had 1.01% ^19^. This variation in results may be due to the number of samples obtained and the sampling areas.

The sociodemographic characteristics that had a higher seropositivity for these parasitic zoonoses were age and occupation. We found a higher frequency of IgG type antibodies against cystic echinococcosis and *T. solium* cysticercosis in adults and older adults, respectively, compared to human fascioliasis, which was found mainly in adolescents and older adults. The increase in cystic echinococcosis and *T. solium* cysticercosis seropositivity with age found by our study is consistent with that found by previous studies ^31^^,^^17^. The most affected occupational groups for these zoonoses were housewives and farmers; a higher cystic echinococcosis seropositivity in housewives has been reported by other studies ^32^. Likewise, the housing material, source of water for human consumption, consumption of unboiled water and defecating outdoors presented a difference in the seropositivity of human fascioliasis, where poverty and deficient sanitary conditions allow the perpetuation of this disease; this has been found by previous studies ^8^^,^^26^^,^^33^.

The habits and activities of the settlers in the studied areas that showed a difference in the seropositivity of these zoonoses were the raising of animals such as pigs and sheep for cystic echinococcosis; goats, cows and sheep for human fascioliasis, and the raising of pigs in free range for *T. solium* cysticercosis. These animals act as hosts in the biological cycle of zoonotic parasites. In this regard, Ghatee *et al.*^34^ found that the population density of sheep, cattle and goats significantly influences the distribution of cystic echinococcosis. In addition, there was a relationship between the higher frequency of *T. solium* cysticercosis and the slaughter of pigs in the peridomicile. This finding could be explained by the fact that raising pigs in unsanitary conditions and consuming their meat without proper inspection may cause greater exposure to *T. solium*^35^. On the other hand, dogs are used for raising cattle, sheep and goats in the countryside. This means that non-dewormed dogs that consume raw viscera from animals slaughtered in the peridomicile are a primary source of cystic echinococcosis infection for humans and animals, which has been demonstrated by our study since we found a difference in the frequency of cases between having non-dewormed dogs and the slaughter of animals in the peridomicile with cystic echinococcosis.

Parasitic zoonoses can be acquired by consuming water and plants contaminated with eggs of *E. granulosus*, *T. solium*, or metacercariae of *F. hepatica*. This fact could explain our results, which show that consuming raw vegetables in juices, extracts and emollients had a difference in the seropositivity of cystic echinococcosis and human fascioliasis. This finding has been reported by previous studies with similar results ^11^^,^^35^.

We found a statistically significant difference in the frequency of cases between weight loss and chest pain with cystic echinococcosis seropositivity. Himsawi *et al*. found that weight loss is associated with cystic echinococcosis ^20^. In addition, another study found an increased frequency of abdominal pain and chronic cough ^36^, which is similar to our findings. Symptoms such as headache, dizziness and nausea had higher *T. solium* cysticercus seropositivity as shown in previous studies ^37^^,^^38^. However, although cysticercosis is associated with epilepsy, we did not identify a difference in case frequency. This finding could be attributed to the fact that we only evaluated data focused on the detection of circulating IgG-type antibodies, which did not allow us to determine neurocysticercosis. A significant difference was found between the frequency of fascioliasis cases and the decrease in weight; which has also been reported by Orfanos *et al*. ^39^ in a study carried out in children from three provinces of Cajamarca. On the other hand, abdominal pain and fever were frequent symptoms in cases with seropositivity to fascioliasis, which is consistent with previous research ^40^^,^^41^.

Likewise, we found that participants with a family history had a higher frequency of the three parasitosis, which has been previously reported by Carmona *et al*^42^. This finding could be due to the fact that people living together in the same environment and sharing the same food and habits could facilitate the transmission of these zoonoses. Therefore, it is recommended to screen the whole family once a case of these etiologies is confirmed. These findings demonstrate the importance of early detection in order to start effective treatment as well as to avoid complications, thus improving public health.

It is important to point out that the main limitation of our study is the use of secondary data, which was obtained previously as part of surveillance activities that do not have a definition established in any manual or guide related to the collection instrument; however, most of the data are clear and have reduced subjectivity. Also, due to the very nature of the study regarding secondary data, it was not possible to include other variables that could be of epidemiological interest, such as the variable “Occupation”, which was collected in an open form in the instrument, and was categorized by the authors in an *ad hoc* manner according to affinity and similarity of the responses. Additionally, the data collected through the epidemiological files were used to establish, in an exploratory manner, a difference in the number of cases identified for each of the parasitic infections. Another important limitation is the representativeness of the analyzed data, given the limited number of subjects screened per region, as well as the non-probabilistic selection made. These data collection limitations were caused by logistical difficulties, since the sampling points were located in rural areas far from the city. Due to the low representativeness of the sample and the methodological design of the screening process (aspects that affect the external validity of the data), it was not possible to establish prevalence and/or seroprevalence estimators, but only case frequency estimators. These limitations should be considered during the critical reading of this article. However, despite the limitations of the sample and design of this study, it should be noted that no previous similar study with a such a large sample size has been found. For this reason, our results are very useful to understand the distribution of these infections. It should also be considered that these parasitic zoonoses are underreported in Peru because they are neglected diseases frequently found in rural areas with extreme poverty, and the Peruvian Ministry of Health (MINSA) has not implemented mandatory notification for their surveillance, prevention and control.

In conclusion, we were able to obtain a better overview of the distribution and characteristics of these parasitic zoonoses in 13 regions of Peru, through the serological analysis of 7811 samples obtained through epidemiological surveillance activities. Our findings could be a useful tool for decision-makers in different sectors, such as Health, Agriculture and Environment, to define strategic areas and/or policies with prioritization or differentiated focus for the control and prevention of these zoonoses.
