# Supplementary material for: Serological surveillance of parasitic zoonoses in 13 highlands regions of Peru: Period 2016-2019
Source: Rev Peru Med Exp Salud Publica. 2023 Jun 30;40(2):189–99. doi: 10.17843/rpmesp.2023.402.12472 (PMC10953634; doi:10.17843/rpmesp.2023.402.12472)
Supplement: Supplementary material. — Available in the electronic version of the RPMESP. [file rpmesp-40-02-12472-s001.docx]

**MATERIAL SUPLEMENTARIO**


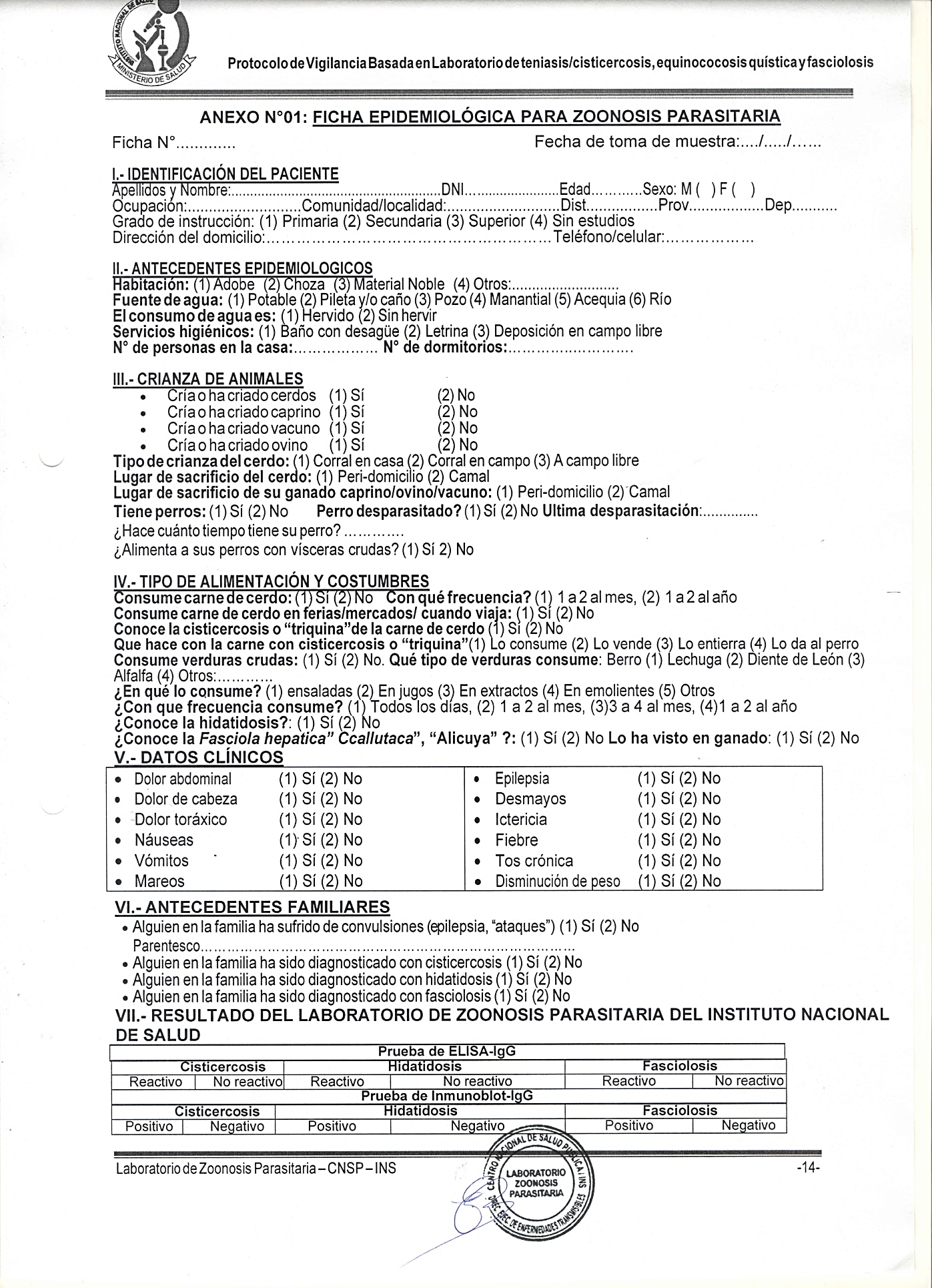


**Figura S1**. Ficha epidemiológica para zoonosis parasitarias que fue empleada para la recolección de datos en actividades de vigilancia. (Fuente: Anexo de la Nota Informativa N° 091-2015-LZP-DEET-CNSP/INS del Instituto Nacional de Salud)

**Tabla S1**. Adecuación/Categorización realizada, para fines del presente artículo, de los ítems originales de la ficha epidemiológica para zoonosis parasitarias sobre las características sociodemográficas.

| **SEGÚN FICHA ORIGINAL** | | | **ADECUACIÓN / CATEGORIZACIÓN** | **SEGÚN LO PRESENTADO EN EL ARTÍCULO** | |
| --- | --- | --- | --- | --- | --- |
| **Sección** | **Item** | **Valores** |  | **Variable** | **Valores** |
| I.- Identificación del Paciente | Edad | Abierto (Sin categoría) | Sí, se adaptó el nombre de la variable y se agrupó según etapa de vida | Grupo Etario | Niños (5 -11 años), Adolescente (12 -17 años), Jóvenes (18 - 29 años), Adulto (30 - 59 años), Adulto mayor (60 a más) |
| I.- Identificación del Paciente | Sexo | Masculino, Femenino | No | Sexo | Femenino, Masculino |
| I.- Identificación del Paciente | Ocupación | Abierto (Sin categoría) | Sí, se categorizó por conveniencia considerando la similitud de respuestas | Ocupación | Estudiante, Salariados/comercio, Ama de casa/limpieza, Agropecuario, No registrado |
| I.- Identificación del Paciente | Grado de Instrucción | Primaria, Secundaria, Superior, Sin instrucción | No | Grado de instrucción | Primaria, Secundaria, Superior, Sin instrucción |
| II.- Antecedentes Epidemiológicos | Habitación | Adobe Choza Material noble Otros | Sí, solo se adaptó el nombre de la variable. | Material de vivienda | De adobe, De choza, De material noble, Otros |
| II.- Antecedentes Epidemiológicos | Fuente de agua | Potable, Pileta y/o caño, Pozo, Manantial, Acequia, Rio | No | Fuente de agua para consumo humano | Potable, Pileta y/o caño, Pozo, Manantial, Acequia, Río |
| II.- Antecedentes Epidemiológicos | El consumo de agua es | Hervido Sin hervir | Sí, se adaptó el nombre de la variable de "El consumo de agua es" a "Consume agua hervida", las respuestas originales de "Hervido" se consideró como "Sí", las respuestas originales "Sin hervir" se consideró como "No", quienes señalaron ambas respuestas originales a la vez fueron considerados como "A veces hervida o no hervida". | Consume agua hervida | Sí, No, A veces hervida o no hervida |
| II.- Antecedentes Epidemiológicos | Servicios higiénicos | Baño con desagüe, Letrina, Deposiciones en campo libre | No | Servicios higiénicos | Baño con desagüe, Letrina, Deposiciones en campo libre |
| I.- Identificación del Paciente | Dep | Abierto (Sin categoría) | Sí, se adaptó el nombre de la variable, se consideraron solo las regiones en las que se ejecutaron actividades de vigilancia y se aplicó la ficha. | Departamento de procedencia | Lima Provincias, Huánuco, Junín, Apurímac, Cusco, Ayacucho, Huancavelica, Cajamarca, Arequipa, Moquegua, Tacna, Puno, Cerro de Pasco |

**Tabla S2**. Adecuación/Categorización realizada, para fines del presente artículo, de los ítems originales de la ficha epidemiológica para zoonosis parasitarias sobre la crianza de animales, faenamiento y prácticas de riesgo.

| **SEGÚN FICHA ORIGINAL** | | | **ADECUACIÓN / CATEGORIZACIÓN** | **SEGÚN LO PRESENTADO EN EL ARTÍCULO** | |
| --- | --- | --- | --- | --- | --- |
| **Sección** | **Item** | **Valores** |  | **Variable** | **Valores** |
| III.- Crianza de Animales | Cría o ha criado cerdos | Sí, No | Sí, se unificó para presentación y adaptó el nombre de la variable | Animales que cría o ha criado | Cerdos, Ganado caprino, Ganado vacuno, Ganado ovino, Perros, |
|  | Cría o ha criado caprino | Sí, No |  |  |  |
|  | Cría o ha criado vacuno | Sí, No |  |  |  |
|  | Cría o ha criado ovino | Sí, No |  |  |  |
|  | Tiene perros | Sí, No |  |  |  |
| III.- Crianza de Animales | Tipo de crianza de cerdo | Corral en casa, Corral en campo, A campo libre | No | Tipo de crianza del cerdo | En corral en casa, En corral en campo, A campo libre |
| III.- Crianza de Animales | Lugar de sacrificio del cerdo | Peri-Domicilio, Camal | No | Lugar de sacrificio del cerdo | Peri domicilio, Camal |
| III.- Crianza de Animales | Lugar de sacrificio de su ganado caprino/ovino/vacuno | Peri-Domicilio, Camal | Sí, se adaptó el nombre de la variable | Lugar de sacrificio de ovino/bovino/caprino | Peri domicilio, Camal |
| III.- Crianza de Animales | Alimenta a sus perros con vísceras crudas? | Sí, No | No | Alimenta a sus perros con vísceras crudas | No, Sí |
| III.- Crianza de Animales | Perro desparasitado | Sí, No | No | Perro desparasitado | No, Sí |
| IV.- Tipo de Alimentación y Costumbres | ¿Conoce la Fasciola hepática "Ccallutaca", "Alicuya"? | Sí, No | Sí, se adaptó el nombre de la variable | Conocimientos sobre la enfermedad Fasciolosis | No, Sí |
| IV.- Tipo de Alimentación y Costumbres | ¿Conoce de hidatidosis? | Sí, No | Sí, se adaptó el nombre de la variable | Conocimientos sobre la enfermedad Equinococosis | No, Sí |
| IV.- Tipo de Alimentación y Costumbres | Conoce la cisticercosis o "triquina" de la cane de cerdo | Sí, No | Sí, se adaptó el nombre de la variable | Conocimientos sobre la enfermedad Cisticercosis | No, Sí |
| IV.- Tipo de Alimentación y Costumbres | Que hace con la carne con cisticercosis o "triquina" | Lo consume, Lo vende, Lo entierra, Lo da al perro | Sí, se adaptó el nombre de la variable | Que hace con la carne con cisticercosis | Lo consume, Lo vende, Lo entierra, Lo da al perro |

**Tabla S3**. Adecuación/Categorización realizada, para fines del presente artículo, de los ítems originales de la ficha epidemiológica para zoonosis parasitarias sobre el consumo de alimentos y bebidas de riesgo para las zoonosis parasitarias.

| **SEGÚN FICHA ORIGINAL** | | | **ADECUACIÓN / CATEGORIZACIÓN** | **SEGÚN LO PRESENTADO EN EL ARTÍCULO** | |
| --- | --- | --- | --- | --- | --- |
| **Sección** | **Item** | **Valores** |  | **Variable** | **Valores** |
| IV.- Tipo de Alimentación y Costumbres | Consume carne de cerdo | Sí, No | Sí, se unificó para presentación y adaptó el nombre de la variable | Condiciones de riesgo en la alimentación | Consume carne de cerdo, Consume verduras crudas |
|  | Consume verduras crudas | Sí, No |  |  |  |
| IV.- Tipo de Alimentación y Costumbres | Con qué frecuencia? | De uno a dos veces al mes, De uno a dos veces al año | Sí, se adaptó el nombre de la variable | Frecuencia de consumo de carne de cerdo | De uno a dos veces al mes, De uno a dos veces al año |
| IV.- Tipo de Alimentación y Costumbres | ¿Con que frecuencia consume? | Todos los días, 1 a 2 al mes, 3 a 4 al mes, 1 a 2 al año | Sí, se adaptó el nombre de la variable | Frecuencia de consumo de verduras | Todos los días, De uno a dos veces al mes, De tres a cuatro veces al mes, De uno a dos veces al año |
| IV.- Tipo de Alimentación y Costumbres | Que tipo de verduras consume | Berro, Lechuga, Diente de león, Alfalfa, Otros | Sí, se adaptó el nombre de la variable | Tipo de verduras que consume | Berro, Lechuga, Diente de león, Alfalfa, Otros |
| IV.- Tipo de Alimentación y Costumbres | ¿En que lo consume? | Ensaladas, En jugo, En extractos, En emolientes, Otros | Sí, se adaptó el nombre de la variable | Forma de consumo de verduras | En ensaladas, En jugo, En extractos, En emolientes, Otros |

**Tabla S4**. Adecuación/Categorización realizada, para fines del presente artículo, de los ítems originales de la ficha epidemiológica para zoonosis parasitarias sobre las características clínicas y epidemiológicas.

| **SEGÚN FICHA ORIGINAL** | | | **ADECUACIÓN / CATEGORIZACIÓN** | **SEGÚN LO PRESENTADO EN EL ARTÍCULO** | |
| --- | --- | --- | --- | --- | --- |
| **Sección** | **Item** | **Valores** |  | **Variable** | **Valores** |
| V.- Datos Clínicos | Dolor abdominal | Sí, No | Sí, se unificó para presentación y adaptó el nombre de la variable | Signos y síntomas | Disminución de peso, Dolor abdominal, Dolor de cabeza, Dolor toráxico, Epilepsia, Fiebre, Ictericia, Mareos, Nauseas, Tos crónica, Vómitos |
|  | Dolor de cabeza | Sí, No |  |  |  |
|  | Dolor toráxico | Sí, No |  |  |  |
|  | Náuseas | Sí, No |  |  |  |
|  | Vómitos | Sí, No |  |  |  |
|  | Mareos | Sí, No |  |  |  |
|  | Epilepsia | Sí, No |  |  |  |
|  | Desmayos | Sí, No |  |  |  |
|  | Ictericia | Sí, No |  |  |  |
|  | Fiebre | Sí, No |  |  |  |
|  | Tos crónica | Sí, No |  |  |  |
|  | Disminución de peso | Sí, No |  |  |  |
| VI.- Antecedentes Familiares | Alguien en la familia ha sido diagnosticado con cisticercosis | Sí, No | Sí, se unificó para presentación y adaptó el nombre de la variable | Antecedentes familiares | Equinococosis, Cisticercosis, Fascioliasis |
|  | Alguien en la familia ha sido diagnosticado con hidatidosis | Sí, No |  |  |  |
|  | Alguien en la familia ha sido diagnosticado con fasciolosis | Sí, No |  |  |  |
