# Supplementary material for: Serological surveillance of parasitic zoonoses in 13 highlands regions of Peru: Period 2016-2019
Source: Rev Peru Med Exp Salud Publica. 2023 Jun 30;40(2):189–99. doi: 10.17843/rpmesp.2023.402.12472 (PMC10953634; doi:10.17843/rpmesp.2023.402.12472)
Supplement: Translation

# Translation: Vigilancia serológica de la zoonosis parasitaria en 13 regiones de la sierra del Perú: Periodo 2016-2019

**Authors:** Isidro Antitupa, Nury Jakeline Vargas-Mayuri, Jhon Vicent Mayo, Luis Arturo Estares-Porras, William Marcelino Quispe Paredes, Elizabeth Luz Sánchez, Gilmer Solis-Sánchez

**Affiliations:** 1 Laboratorio de Referencia Nacional de Metaxénicas y Zoonosis Parasitarias, Instituto Nacional de Salud, Lima, Perú.; 2 Dirección de Prevención y Control de Enfermedades Metaxénicas y Zoonosis de la Dirección General de Intervenciones Estratégicas en Salud Pública, Ministerio de Salud, Lima, Perú.; 3 Universidad Científica del Sur, Lima, Perú.

**Keywords:** Cisticercosis, Equinococosis, Fascioliasis, ELISA, Estudios Seroepidemiológicos, Vigilancia en Salud Pública, Zoonosis, Perú

## Abstract

**Objetivos.:**

Determinar la seropositividad a anticuerpos anti-IgG por infección de *Echinococcus granulosus, Fasciola hepatica* y cisticerco de *Taenia solium* y describir las características de los infectados en 13 regiones de la sierra peruana entre 2016 y 2019.

**Materiales y métodos.:**

Estudio observacional transversal, que analizó 7811 fichas epidemiológicas de la vigilancia basada en laboratorio de las zoonosis parasitarias del periodo 2016-2019. El diagnóstico se realizó mediante la detección de anticuerpos tipo IgG anti *E. granulosus, F. hepatica* y cisticerco de *T. solium* utilizando antígenos nativos mediante el ensayo inmunoabsorbente ligado a enzimas (ELISA) e Inmunoblot. La diferencia en la frecuencia de casos de estas zoonosis según características identificadas se realizó mediante la prueba chi-cuadrado de Pearson y prueba exacta de Fisher.

**Resultados.:**

Se determinó una seropositividad de 7,9% para fascioliasis, 4,9% para equinococosis quística, y 2,3% para cisticerco de *T. solium*. Estas frecuencias fueron mayores en Cerro de Pasco para equinococosis quística (24,5%), en Ayacucho para cisticerco de *T. solium* (4,5%) y en Puno para fascioliasis (40,6%). Entre las características sociodemográficas, se encontró una diferencia estadísticamente significativa en la frecuencia de casos para todas las zoonosis según grupo etario, ocupación, y región de residencia. Además, se encontró diferencia con el consumo de verduras en emolientes, y entre las características clínico-epidemiológicas con tener antecedentes familiares de las zoonosis parasitarias.

**Conclusiones.:**

A partir de las 7811 muestras evaluadas, se encontró que estas zoonosis parasitarias están distribuidas en 13 regiones de la sierra del Perú, ocasionando un problema de salud importante, con frecuencias que varían según diversas características.

## INTRODUCCIÓN

Las zoonosis parasitarias son enfermedades desatendidas, que son transmitidas naturalmente desde animales vertebrados al ser humano, siendo las de mayor importancia, las zoonosis causadas por helmintos como *Fasciola hepatica*, *Echinococcus granulosus* y *Taenia solium*^1^. En el humano, la infección por cisticerco de *T. solium* afecta principalmente el sistema nervioso central (SNC), la infección por *F. hepatica* afecta los conductos biliares del hígado, y la infección por *E. granulosus* afecta el hígado y el pulmón. Todas estas parasitosis tisulares, son asintomáticas hasta la fase crónica de la enfermedad, donde los principales signos y síntomas empiezan a manifestarse, generando un deterioro significativo de la salud de las personas ^2^.

La endemicidad de estas zoonosis está distribuida principalmente en África, Asia, Sur de Europa y América del Sur ^3^^-^^5^. En Perú, se encontraron seroprevalencias en humanos, de hasta 20% de equinococosis quística en Cerro de Pasco ^6^, 24% de cisticerco de *T. solium* en Saylla (Cusco) ^7^ y hasta 31% de fascioliasis en Puno ^8^, que son zonas rurales y ganaderas de la sierra central y sierra sur. Se estima que, en el Perú, la carga de la enfermedad por años de vida ajustados por discapacidad (AVAD) para equinococosis quística humana es de 1139 años con un costo total anual de 2,420,348 dólares americanos ^9^; sin embargo, aún no se han realizado estimaciones de AVAD para cisticerco de *T. solium* y fascioliasis humana.

Los principales factores de riesgo asociados con equinococosis quística humana, que se reportaron en población peruana, son la exposición a perros infectados, contacto con ganado infectado y consumo de alimentos contaminados con huevos de *E. granulosus* que, es la forma infectante del parásito ^10^. La fascioliasis humana está asociada con determinantes como la exposición a ganado infectado y el consumo de vegetales crudos expuestos a agua de riego contaminados con metacercarias de *F. hepatica* que, es la forma infectante del trematodo ^11^. Por otro lado, se encontraron factores de riesgo asociados con cisticerco de *T. solium*, tales como el consumo de carne de cerdo cruda o insuficientemente cocida contaminada con larvas de cisticerco de *T. solium*, consumo de alimentos y agua contaminada con heces de personas con teniasis a través de la transmisión directa de los huevos de *T. solium* que, es la forma infectante para el cisticerco de *T. solium*^12^. Además, existen determinantes comunes para las tres zoonosis como los hábitos higiénicos deficientes, y condiciones socioeconómicas desfavorables, donde resalta la falta de acceso a servicios de salud adecuados, educación y saneamiento básico ^10^^-^^12^.

Existen estudios de distribución de las zoonosis a nivel nacional focalizados en niños en edad escolar, sin embargo, no se han determinado las comunidades de alto riesgo de las zoonosis en el territorio peruano ni la magnitud de la enfermedad en la población general. Por este motivo, el presente estudio buscó determinar la seropositividad a anticuerpos anti-IgG por infección de *E. granulosus*, *F. hepatica* y cisticerco de *T. solium* y describir las características de los infectados en 13 regiones de la sierra peruana entre 2016 y 2019.

**MENSAJE CLAVE**

Motivación para realizar el estudio. Conocer las características y distribución de las principales zoonosis parasitarias en el Perú y generar data para la toma de decisiones en la vigilancia, prevención y control.

Principales hallazgos. En los tres grupos de edad analizados, la enfermedad neurológica crónica, la enfermedad renal, la enfermedad hepática y el cáncer conllevan a un alto riesgo de morir por la COVID-19, en pacientes hospitalizados en Lima y Callao. El riesgo de mortalidad asociada con comorbilidades fue mayor en pacientes de 18 a 29 años.

Implicancias. Este estudio contribuye a identificar los grupos de pacientes con el mayor riesgo de muerte por la COVID-19, según grupo de edad y tipo de comorbilidad.

## MATERIALES Y MÉTODOS

### Diseño del estudio

Se realizó un estudio observacional de enfoque cuantitativo de diseño transversal, en el cual, se analizaron las fichas epidemiológicas de las zoonosis parasitarias y resultados obtenidos en las actividades de la vigilancia basada en laboratorio para la identificación de zoonosis parasitaria, ejecutadas por el Laboratorio de Referencia Nacional de Zoonosis Parasitaria del Instituto Nacional de Salud (LRNZOP-INS) entre los años 2016 y 2019.

### Población de estudio

Se evaluaron las fichas epidemiológicas de pobladores que participaron en las actividades de vigilancia epidemiológica basada en laboratorio entre 2016 y 2019, quienes provenían de 13 regiones de la sierra del Perú (Apurímac, Arequipa, Ayacucho, Cajamarca, Cerro de Pasco, Cusco, Huancavelica, Huánuco, Junín, Lima Provincias, Moquegua, Puno y Tacna), que se encuentran entre los 1800 y los 4100 metros sobre el nivel del mar, con un estimado de pobreza total entre 23 y 44% ^13^, y se caracterizan por tener como principal actividad económica a la ganadería y agricultura.

### Muestra y selección de participantes

En el análisis, se incluyó a la totalidad de fichas epidemiológicas disponibles que fueron realizadas en cada departamento de procedencia. La ejecución de las fichas se basó en el número de pobladores de cada localidad y la capacidad logística de las Direcciones Regionales de Salud (DIRESA) de las regiones que, implementaron la actividad de vigilancia. Los tamizajes se ejecutaron por conveniencia mediante búsqueda proactiva de los participantes en casas e instituciones educativas de cada localidad. El tamizaje consideró a personas mayores de cinco años y con residencia permanente en zonas endémicas, mientras que las personas que presentaron otras patologías por laboratorio fueron excluidas. Para el análisis, se excluyeron las fichas que tenían datos incompletos.

### Recolección de datos

Los datos analizados provenían de información recolectada mediante la ficha epidemiológica de las zoonosis parasitarias: equinococosis quística, fascioliasis y cisticerco de *T. solium* (Material Suplementario) y los resultados de las pruebas serológicas de cada uno de los participantes tamizados registrados en el sistema NETLAB.

Las fichas epidemiológicas analizadas fueron elaboradas por el LRNZOP-INS para la realización de las actividades de vigilancia. Estas fichas han sido revisadas previamente por expertos técnicos, fueron aplicadas por personal capacitado, y se encontraban almacenadas en el LRNZOP-INS, como una herramienta para la elaboración de informes técnicos sobre la situación epidemiológica de estas enfermedades parasitarias.

### Datos serológicos

Posterior al llenado de la ficha epidemiológica, se obtuvo una muestra de 5 mL de sangre venosa, que fue empleada para el diagnóstico serológico por el método de ELISA-IgG e Inmunoblot-IgG. Cabe precisar que, ambos kits de diagnóstico fueron producidos en el LRNZOP-INS. El método de Inmunoblot para equinococosis quística tiene una sensibilidad de 95% y especificidad de 100% para las bandas de 8 kDa, 16 kDa, y 21 kDa, empleando antígenos del líquido hidatídico de *E. granulosus*. Por otro lado, el Inmunoblot para cisticerco de *T. solium* tiene una sensibilidad de 93% y especificidad de 100% para las bandas de 13 kDa, 14 kDa, 17 kDa, 18 kDa, 23 kDa, 24 kDa, 31 kDa, y 35 kDa, empleando antígenos del líquido vesicular del cisticerco de *T. solium*. Finalmente, el Inmunoblot para fascioliasis tiene una sensibilidad de 91% y especificidad de 99%, empleando antígenos purificados de 27-28 kDa desde los productos de secreción/excreción de *F. hepatica*^14^^,^^15^.

Primero, se realizó el tamizaje serológico de cada etiología, empleando el método de ELISA-IgG para equinococosis quística, fascioliasis y cisticerco de *T. solium,* que fueron ejecutadas en los Laboratorios de Referencia Regional (LRR) de cada DIRESA. Luego, se realizó la confirmación diagnóstica empleando el método de Inmunoblot-IgG para equinococosis quística, fascioliasis, y cisticerco de *T. solium* de las muestras con resultado REACTIVO en el método de ELISA-IgG, que fueron ejecutadas en el LRNZOP-INS.

### Variables

La variable dependiente corresponde al resultado categórico (positivo, negativo) del diagnóstico serológico de las zoonosis según tipo de parasitosis evaluada. Las variables independientes corresponden a los datos obtenidos mediante las fichas epidemiológicas que han sido materia de interés en otros estudios para este tipo de infecciones ^11^^,^^16^^-^^20^. Estas variables comprendían información sobre las características sociodemográficas, crianza de animales, faenamiento y prácticas de riesgo, consumo de alimentos y bebidas de riesgo, y características clínicas y epidemiológicas.

Los nombres de algunas variables evidencian una adaptación de los nombres de los ítems de las fichas para una adecuada presentación. Algunos ítems de la ficha original incorporaron la alternativa «otros» para permitir una recolección exhaustiva de respuestas que fueron consideradas, según criterio del equipo del LRNZOP-INS, como poco frecuentes al momento de la elaboración de la ficha. Entre estas variables, se encuentra el «Material de vivienda» (que podría abordar piedra con barro, cartón, piedra o sillar con cal, etc.), así como «Tipo de verduras que consume» y «Forma de consumo de verduras». Las características de las adecuaciones/agrupaciones de las variables se presentan en el Material Suplementario.

Las características sociodemográficas evaluadas fueron edad, sexo, ocupación, grado de instrucción, material de vivienda, fuente de agua para consumo humano, consumo de agua hervida, tipo de servicios higiénicos, y departamento de procedencia. Características relacionadas con la crianza de animales, faenamiento y prácticas de riesgo. Respecto a las prácticas de riesgo, se registraron los datos del tipo de animales que cría o ha criado, tipo de crianza y lugar de sacrificio del cerdo, lugar de sacrificio de ovinos/bovinos/caprinos, alimentación a sus perros con vísceras crudas, desparasitación de sus perros y manejo de la carne con cisticercosis. En cuanto a la categorización de las variables, la edad fue categorizada según etapa de vida, que es una clasificación ampliamente utilizada en sistemas de salud, mientras que la ocupación fue categorizada por conveniencia considerando la similitud y afinidad de respuestas obtenidas de forma abierta en la ficha epidemiológica.

En cuanto a las características de consumo de alimentos y bebidas de riesgo, se registró información sobre los alimentos que consume, la frecuencia de consumo de carne de cerdo y verduras, tipo de verduras que consume y forma de consumo. Las características clínicas y epidemiológicas evaluadas comprendieron antecedentes semiológicos personales (desmayo, disminución de peso, dolor abdominal, dolor de cabeza, dolor torácico, epilepsia, fiebre, ictericia, mareos, náuseas, tos crónica y vómitos), y antecedentes familiares de equinococosis, cisticercosis y fascioliasis.

### Análisis estadístico

El análisis de datos fue llevado a cabo con el paquete estadístico Stata v17.0. (Stata Corporation, College Station, Texas, USA), para realizar estimaciones descriptivas de las variables de estudio obteniendo frecuencias absolutas y relativas. De manera exploratoria, se identificó la diferencia en la proporción de cada infección según todas las características de estudio mediante la prueba chi-cuadrado de Pearson o Exacta de Fisher (según la regla de Cochran). Estas pruebas se ejecutaron considerando un nivel de significancia de 0,05.

### Aspectos éticos

El presente estudio corresponde a una evaluación de datos secundarios los cuales fueron recolectados durante actividades de vigilancia epidemiológica en el marco del «Protocolo de Vigilancia Basada en Laboratorio de las Zoonosis Parasitarias (Teniosis/Cisticercosis, Equinococosis quística, y Fasciolosis)» aprobado el 2015 por el Centro Nacional de Salud Pública del INS.

Previo a la obtención de información clínica y de laboratorio, según lo establecido en el citado protocolo, cada participante brindó su autorización mediante consentimiento y/o asentimiento informado

## RESULTADOS

De las 9389 fichas epidemiológicas identificadas, se excluyeron 1578 debido a falta de información. Finalmente, se incluyó un total de 7811 fichas epidemiológicas obtenidas de pobladores de 13 regiones de la sierra del Perú (Figura 1).

**Figura 1:** Flujograma de conformación las fichas epidemiológicas analizadas.

En la Tabla 1, se describen las características sociodemográficas de la población estudiada, donde el 36,7% eran adultos, el 65% eran varones, y el 41,4% eran estudiantes. Asimismo, la mayoría de los participantes tenía solo estudios primarios (44,4%), adobe como material de pared de la vivienda (85,2%), abastecimiento de agua mediante pileta o caño (52,3%), consumía agua hervida (86,1%), contaba con baño con desagüe (50,5%) y eran de Apurímac (26,2%).

**Tabla 1:** Características sociodemográficas de la población estudiada en 13 regiones de la sierra del Perú, 2016-2019.

Características sociodemográficas	Total de fichas evaluadas		Equinococosis quística positiva	Valor de p	Cisticercosis positiva	Valor de p	Fascioliasis positiva	Valor de p
	n / N	%	n/N (%)		n/N (%)		n/N (%)	
Grupo Etario								
Niños (5 -11 años)	1241 / 7811	15,9	42 / 1241 (3,4)	0,031^a^	10 / 1241 (0,8)	<0,001^a^	96 / 1241 (7,7)	<0,001^a^
Adolescente (12 -17 años)	1774 / 7811	22,7	77 / 1774 (4,3)		25 / 1774 (1,4)		172 / 1774 (9,7)	
Jóvenes (18 - 29 años)	974 / 7811	12,5	53 / 974 (5,4)		24 / 974 (2,5)		76 / 974 (7,8)	
Adulto (30 - 59 años)	2866 / 7811	36,7	157 / 2866 (5,5)		83 / 2866 (2,9)		178 / 2866 (6,2)	
Adulto mayor (60 a más)	956 / 7811	12,2	52 / 956 (5,4)		39 / 956 (4,1)		93 / 956 (9,7)	
Sexo								
Femenino	2730 / 7811	35,0	129 / 2730 (4,7)	0,647^a^	51 / 2730 (1,9)	0,053^a^	214 / 2730 (7,8)	0,934^a^
Masculino	5081 / 7811	65,0	252 / 5081 (5,0)		130 / 5081 (2,6)		401 / 5081 (7,9)	
Ocupación								
Estudiante	3230 / 7811	41,4	136 / 3230 (4,2)	0,019^a^	37 / 3230 (1,1)	<0,001^a^	280 / 3230 (8,7)	<0,001^a^
Salariados/comercio	671 / 7811	8,6	23 / 671 (3,4)		20 / 671 (3,0)		22 / 671 (3,3)	
Ama de casa/limpieza	1635 / 7811	20,9	93 / 1635 (5,7)		67 / 1635 (4,1)		94 / 1635 (5,7)	
Agropecuario	2248 / 7811	28,8	128 / 2248 (5,7)		56 / 2248 (2,5)		219 / 2248 (9,7)	
No registrado	27 / 7811	0,3	1 / 27 (3,7)		1 / 27 (3,7)		0 / 27 (0,0)	
Grado de instrucción								
Primaria	3278 / 7389	44,4	149 / 3278 (4,5)	0,554^a^	81 / 3278 (2,5)	0,194^a^	262 / 3278 (8,0)	0,034^a^
Secundaria	3242 / 7389	43,9	159 / 3242 (4,9)		68 / 3242 (2,1)		258 / 3242 (8,0)	
Superior	604 / 7389	8,2	28 / 604 (4,6)		10 / 604 (1,7)		28 / 604 (4,6)	
Sin instrucción	265 / 7389	3,6	17 / 265 (6,4)		10 / 265 (3,8)		21 / 265 (7,9)	
Material de vivienda								
De adobe	6144 / 7210	85,2	272 / 6144 (4,4)	0,519^a^	152 / 6144 (2,5)	0,064^a^	502 / 6144 (8,2)	<0,001^a^
De choza	126 / 7210	1,7	9 / 126 (7,1)		5 / 126 (4,0)		14 / 126 (11,1)	
De material noble	802 / 7210	11,1	35 / 802 (4,4)		9 / 802 (1,1)		31 / 802 (3,9)	
Otros	138 / 7210	1,9	7 / 138 (5,1)		4 / 138 (2,9)		5 / 138 (3,6)	
Fuente de agua para consumo humano								
Potable	2427 / 6486	37,4	117 / 2427 (4,8)	0,200^a^	65 / 2427 (2,7)	0,304^a^	196 / 2427 (8,1)	<0,001^a^
Pileta y/o caño	3393 / 6486	52,3	145 / 3393 (4,3)		77 / 3393 (2,3)		230 / 3393 (6,8)	
Pozo	301 / 6486	4,6	10 / 301 (3,3)		9 / 301 (3,0)		37 / 301 (12,3)	
Manantial	245 / 6486	3,8	11 / 245 (4,5)		8 / 245 (3,3)		22 / 245 (9,0)	
Acequia	49 / 6486	0,8	5 / 49 (10,2)		0 / 49 (0,0)		6 / 49 (12,2)	
Río	71 / 6486	1,1	1 / 71 (1,4)		4 / 71 (5,6)		0 / 0 (0,0)	
Consume agua hervida								
Sí	6212 / 7213	86,1	305 / 6212 (4,9)	0,027†	146 / 6212 (2,4)	0,857^a^	457 / 6212 (7,4)	0,024†
No	855 / 7213	11,9	33 / 855 (3,9)		18 / 855 (2,1)		72 / 855 (8,4)	
A veces hervida o no hervida	146 / 7213	2,0	1 / 146 (0,7)		4 / 146 (2,7)		19 / 146 (13,0)	
Servicios higiénicos								
Baño con desagüe	3592 / 7111	50,5	169 / 3592 (4,7)	0,445^a^	79 / 3592 (2,2)	0,353^a^	207 / 3592 (5,8)	<0,001^a^
Letrina	2987 / 7111	42,0	123 / 2987 (4,1)		73 / 2987 (2,4)		262 / 2987 (8,8)	
Deposiciones en campo libre	532 / 7111	7,5	21 / 532 (3,9)		17 / 532 (3,2)		70 / 532 (13,2)	
Departamento de procedencia								
Lima Provincias	1187 / 7811	15,2	73 / 1187 (6,1)	<0,001^a^	16 / 1187 (1,3)	<0,001^a^	155 / 1187 (13,1)	<0,001^a^
Huánuco	903 / 7811	11,6	17 / 903 (1,9)		17 / 903 (1,9)		78 / 903 (8,6)	
Junín	528 / 7811	6,8	28 / 528 (5,3)		9 / 528 (1,7)		29 / 528 (5,5)	
Apurímac	2047 / 7811	26,2	67 / 2047 (3,3)		36 / 2047 (1,8)		121 / 2047 (5,9)	
Cusco	1318 / 7811	16,9	57 / 1318 (4,3)		50 / 1318 (3,8)		91 / 1318 (6,9)	
Ayacucho	969 / 7811	12,4	78 / 969 (8,0)		44 / 969 (4,5)		60 / 969 (6,2)	
Huancavelica	197 / 7811	2,5	14 / 197 (7,1)		1 / 197 (0,5)		7 / 197 (3,6)	
Cajamarca	140 / 7811	1,8	0 / 140 (0,0)		0 / 140 (0,0)		28 / 140 (20,0)	
Arequipa	188 / 7811	2,4	7 / 188 (3,7)		2 / 188 (1,1)		29 / 188 (15,4)	
Moquegua	14 / 7811	0,2	0 / 14 (0,0)		0 / 14 (0,0)		0 / 14 (0,0)	
Tacna	133 / 7811	1,7	1 / 133 (0,8)		0 / 133 (0,0)		2 / 133 (1,5)	
Puno	32 / 7811	0,4	1 / 32 (3,1)		0 / 32 (0,0)		13 / 32 (40,6)	
Cerro de Pasco	155 / 7811	2,0	38 / 155 (24,5)		6 / 155 (3,9)		2 / 155 (1,3)	
Total	----	----	381 / 7811 (4,9)		181 / 7811 (2,3)		615 / 7811 (7,9)	

a Prueba Chi Cuadrado de Pearson.

La frecuencia de casos de equinococosis quística, cisticercos de *T. solium* y fascioliasis fue de 4,9%, 2,3% y 7,9%, respectivamente. Además, se encontró que la mayor cantidad de casos de equinococosis quística se obtuvo en adultos (5,5%), para cisticerco de *T. solium* en adultos mayores (4,1%), y de fascioliasis en adolescentes y adultos mayores (9,7%). El grupo ocupacional más afectado por equinococosis quística fueron las amas de casa y agropecuarios (5,7%), para cisticerco de T. solium fueron las amas de casa (4,1%), y los agropecuarios para fascioliasis (9,7%). Se encontró positividad significativa de fascioliasis a predominio de personas con formación primaria y secundaria (8,0%). Asimismo, la mayor frecuencia de fascioliasis se encontró en los participantes que residían en una vivienda de material de chozas (11,1%), así como quienes recibían abastecimiento de agua de pozo (12,3%), y realizaban deposiciones en campo libre (13,2%). Entre las regiones evaluadas, la mayor frecuencia de equinococosis quística se encontró en Cerro de Pasco (24,5%), de cisticercosis en Ayacucho (4,5%), y de fascioliasis en Puno (40,6%) (Tabla 1).

En cuanto a las costumbres y actividades de las personas, se encontró una positividad significativa de equinococosis quística en los participantes que criaban cerdos (5,4%) y ovejas (3,8%); y de fascioliasis en criadores de cabras (7,2%), vacas (6,2%) y ovejas (6,5%). La mayor frecuencia de cisticercosis se encontró en los participantes que criaban cerdos a campo libre (4,1%), y quienes sacrificaban a sus cerdos cerca a sus casas (2,4%). Las personas con perros no desparasitados tuvieron mayor frecuencia de equinococosis quística (6,0%), mientras que los casos de cisticercosis fueron mayores en quienes vendían carne contaminada con cisticerco de *T. solium* (5,7%) (Tabla 2).

**Tabla 2:** Distribución de las zoonosis parasitarias según las características de crianza de animales, faenamiento y prácticas de riesgo en 13 regiones de la sierra del Perú, 2016-2019.

Crianza de animales, faenamiento y prácticas de riesgo	Total de fichas evaluadas		Equinococosis quística positiva n/N (%)	Valor de p	Cisticercosis positiva n/N (%)	Valor de p	Fascioliasis positiva n/N (%)	Valor de p
	n / N	%						
Animales que cría o ha criado								
Cerdos	4022 / 7202	55,8	217/4022 (5,4)	<0,001^a^	99 / 4022 (2,5)	0,415^a^	310 / 4022 (7,7)	0,649^a^
Ganado caprino	6447 / 7121	90,5	283/6447 (4,4)	0,543^a^	147 / 6447 (2,3)	0,877^a^	462 / 6447 (7,2)	<0,001^a^
Ganado vacuno	3583 / 7194	49,8	164/3583 (4,6)	0,597^a^	77 / 3583 (2,1)	0,373^a^	221 / 3583 (6,2)	<0,001^a^
Ganado ovino	4054 / 7209	56,2	153/4054 (3,8)	<0,001^a^	84 / 4054 (2,1)	0,163^a^	263 / 4054 (6,5)	<0,001^a^
Perros	1793 / 7247	24,7	83/1793 (4,6)	0,787^a^	47 / 1793 (2,6)	0,326^a^	117 / 1793 (6,5)	0,069^a^
Tipo de crianza del cerdo								
En corral en casa	1452 / 3423	42,4	82/1452 (2,7)	0,271^a^	27 / 1452 (1,9)	0,001^a^	117 / 1452 (8,1)	0,617^a^
En corral en campo	1140 / 3423	33,3	43/1140 (3,8)		19 / 1140 (1,7)		92 / 1140 (8,1)	
A campo libre	831 / 3423	24,3	29/831 (3,5)		34 / 831 (4,1)		76 / 831 (9,1)	
Lugar de sacrificio del cerdo								
Peridomicilio	2968 / 3263	91,0	96/2968 (3,2)	0,445^a^	71 / 2968 (2,4)	0,022^a^	249 / 2968 (8,4)	0,580^a^
Camal	295 / 3263	9,0	12/295 (4,1)		1 / 295 (0,3)		22 / 295 (7,5)	
Lugar de sacrificio de ovino/bovino/caprino								
Peridomicilio	3558 / 3840	92,7	149/3558 (4,2)	0,957†	97 / 3558 (2,7)	0,040†	319 / 3558 (9,0)	0,204†
Camal	282 / 3840	7,3	12/282 (4,3)		2 / 282 (0,7)		19 / 282 (6,7)	
Alimenta a sus perros con vísceras crudas								
No	2026 / 4879	41,5	104/2026 (5,1)	0,043†	49 / 2026 (2,4)	0,516†	188 / 2026 (9,3)	0,051†
Sí	2853 / 4879	58,5	112/2853 (3,9)		61 / 2853 (2,1)		220 / 2853 (7,7)	
Perro desparasitado								
No	1451 / 4834	30,0	87/1451 (6,0)	<0,001^a^	39 / 1451 (2,7)	0,162^a^	103 / 1451 (7,1)	0,092^a^
Sí	3383 / 4834	70,0	112/3383 (3,3)		69 / 3383 (2,0)		289 / 3383 (8,5)	
Conocimientos sobre la enfermedad:								
Fascioliasis								
No	2715 / 6579	41,3	132/2715 (4,9)	0,027^a^	84/2715 (3,1)	0,001^a^	215/2715 (7,9)	0,939^a^
Sí	3864 / 6579	58,7	145/3864 (3,8)		72 / 3864 (1,9)		304 / 3864 (7,9)	
Equinococosis								
No	1731 / 4959	34,9	83/1731 (4,8)	0,035^a^	58/1731 (3,4)	<0,001^a^	132/1731 (7,6)	0,082^a^
Sí	3228 / 4959	65,1	115/3228 (3,6)		56 / 3228 (1,7)		293/3228 (9,1)	
Cisticercosis								
No	2754 / 5794	47,5	134/2754 (4,9)	0,009^a^	80/2754 (2,9)	0,004^a^	208/2754 (7,6)	0,106^a^
Sí	3040 / 5794	52,5	106/3040 (3,5)		54/3040 (1,8)		265/3040 (8,7)	
Que hace con la carne con cisticercosis								
Lo consume	342 / 3238	10,6	10/342 (2,9)	0,397^a^	12 / 342 (3,5)	0,019^a^	23/342 (6,7)	0,872^a^
Lo vende	123 / 3238	3,8	4/123 (3,3)		7 / 123 (5,7)		9/123 (7,3)	
Lo entierra	2430 / 3238	75,0	114/2430 (4,7)		61 / 2430 (2,5)		192/2430 (7,9)	
Lo da al perro	343 / 3238	10,6	13/343 (3,8)		3 / 343 (0.9)		25/343 (7.3)	

a Prueba Chi Cuadrado de Pearson.

En lo referente a consumo de alimentos y bebidas de riesgo para las zoonosis parasitarias, se encontró mayor positividad para cisticercosis entre los participantes que consumían carne de cerdo (2,9%), sin embargo, no se obtuvo una diferencia estadísticamente significativa. Además, se reportó que los participantes que consumían emolientes y extractos de verduras tenían una mayor frecuencia de fascioliasis y equinococosis quística (p<0,05). Adicionalmente, se evidenció una diferencia en los casos de equinococosis quística por consumo de verduras en jugos (p<0,05) (Tabla 3).

**Tabla 3:** Distribución de las zoonosis parasitarias según el consumo de alimentos y bebidas de riesgo para las zoonosis parasitarias encontradas en 13 regiones de la sierra del Perú, 2016-2019.

Consumo de alimentos y bebidas de riesgo para las zoonosis parasitarias	Total de fichas evaluadas		Equinococosis quística positiva	Valor de p	Cisticercosis positiva	Valor de p	Fascioliasis positiva	Valor de p
	n / n	%	n/N (%)		n/N (%)		n/N (%)	
Condiciones de riesgo en la alimentación								
Consume carne de cerdo	1329 / 4938	26,9	76/1329 (5,7)	0,006^a^	38 / 1329 (2,9)	0,295^a^	114 / 1329 (8,6)	0,173^a^
Consume verduras crudas	568 / 4938	11,5	19/568 (3,3)	0,169^a^	14 / 568 (2,5)	0,815^a^	42 / 568 (7,4)	0,853^a^
Frecuencia de consumo de carne de cerdo								
De uno a dos veces al mes	1849 / 5031	36,8	67/1849 (3,6)	0,199^a^	31 / 1849 (1,7)	0,092^a^	129 / 1849 (7,0)	0,169^a^
De uno a dos veces al año	3182 / 5031	63,2	139/3182 (4,4)		76 / 3182 (2,4)		256 / 3182 (8,0)	
Frecuencia de consumo de verduras								
Todos los días	906 / 5441	16,7	28/906 (3,1)	0,271^a^	15 / 906 (1,7)	0,397^a^	69 / 906 (7,6)	0,102^a^
De uno a dos veces al mes	3076 / 5441	56,5	134/3076 (4,4)		79 / 3076 (2,6)		287 / 3076 (9,3)	
De tres a cuatro veces al mes	1356 / 5441	24,9	64/1356 (4,7)		29 / 1356 (2,1)		99 / 1356 (7,3)	
De uno a dos veces al año	103 / 5441	1,9	5/103 (4,9)		3 / 103 (2,9)		8 / 103 (7,8)	
Tipo de verduras que consume								
Berro	1949 / 5614	34,7	93/1949 (4,8)	0,399^a^	50 / 1949 (2,6)	0,401^a^	155 / 1949 (8,0)	0,708^a^
Lechuga	4275 / 5614	76,1	194/4275 (4,5)	0,582^a^	102 / 4275 (2,4)	0,641^a^	337 / 4275 (7,9)	0,208^a^
Diente de león	112 / 5614	2,0	7/112 (6,3)	0,348^b^	2 / 112 (1,8)	1,000^b^	7 / 112 (6,3)	0,460^a^
Alfalfa	162 / 5614	2,9	5/162 (3,1)	0,392^a^	1 / 162 (0,6)	0,186^b^	10 / 162 (6,2)	0,353^a^
Otros	18 / 5614	0,3	1/18 (5,6)	0,560^b^	1 / 18 (5,6)	0,347^b^	0 / 18 (0,0)	0,393^b^
Forma de consumo de verduras								
En ensaladas	5231 / 5614	93,2	234/5231 (4,5)	0,786^a^	120 / 5231 (2,3)	0,469^a^	429 / 5231 (8,2)	0,538^a^
En jugo	420 / 5614	7,5	36/420 (8,6)	<0,001^a^	10 / 420 (2,4)	0,947^a^	34 / 420 (8,1)	0,972^a^
En extractos	210 / 5614	3,7	17/210 (8,1)	0,009^a^	1 / 210 (0,5)	0,096^b^	4 / 210 (1,9)	0,001^a^
En emolientes	235 / 5614	4,2	4/235 (1,7)	0,037^a^	1 / 235 (0,4)	0,048^a^	6 / 235 (2,6)	0,001^a^
Otros	66 / 5614	1,2	3/66 (4,5)	0,769^b^	3 / 66 (4,5)	0,199^b^	4 / 66 (6,1)	0,534^a^

a Prueba Chi Cuadrado de Pearson.

b Prueba exacta de Fisher.

En cuanto a las características clínicas y epidemiológicas de los pacientes positivos, se destaca que los principales síntomas para equinococosis quística fueron la disminución de peso (4,3%), dolor torácico (4,2%) y dolor abdominal (4,4%). Para la cisticercosis fueron el dolor de cabeza (1,8%), mareos (2,0%), y náuseas (2,2%), mientras que para la fascioliasis fueron la disminución de peso (7,6%), dolor abdominal (8,0%), fiebre (7,9%) e ictericia (7,8%). Respecto a la evaluación de los antecedentes familiares, se encontró una diferencia estadísticamente significativa en la frecuencia de casos (p<0,05) para las tres zoonosis parasitarias (Tabla 4).

**Tabla 4:** Características clínicas y epidemiológicas de las zoonosis parasitarias encontradas en 13 regiones de la sierra del Perú, 2016-2019.

Características clínicas y epidemiológicas	Total de fichas evaluadas		Equinococosis quística positiva	Valor de p	Cisticercosis positiva	Valor de p	Fascioliasis positiva	Valor de p
	n / n	%	n/N (%)		n/N (%)		n/N (%)	
Signos y Síntomas								
Disminución de peso	5540 / 6246	88,7	238/5540 (4,3)	0,005^a^	122 / 5540 (2,2)	0,047^a^	420 / 5540 (7,6)	0,010^a^
Dolor abdominal	3320 / 6235	53,2	146/3320 (4,4)	0,374^a^	71 / 3320 (2,1)	0,224^a^	265 / 3320 (8,0)	0,792^a^
Dolor de cabeza	3306 / 6220	53,2	133/3306 (4,0)	0,018^a^	59 / 3306 (1,8)	0,001^a^	262 / 3306 (7,9)	0,997^a^
Dolor toráxico	5013 / 6184	81,1	213/5013 (4,2)	0,008^a^	111 / 5013 (2,2)	0,161^a^	409 / 5013 (8,2)	0,223^a^
Epilepsia	6189 / 6260	98,9	272/6189 (4,4)	0,770^b^	139 / 6189 (2,2)	0,675^b^	386 / 6189 (6,2)	<0,001^b^
Fiebre	4858 / 6247	77,8	218/4858 (4,5)	0,521^a^	108 / 4858 (2,2)	0,265^a^	384 / 4858 (7,9)	0,986^a^
Ictericia	6049 / 6241	96,9	277/6049 (4,6)	0,787^a^	137 / 6049 (2,3)	0,088^b^	471 / 6049 (7,8)	0,063^a^
Mareos	4968 / 6252	79,5	228/4968 (4,6)	0,912^a^	98 / 4968 (2,0)	<0,001^a^	382 / 4968 (7,7)	0,298^a^
Nauseas	4883 / 6200	78,8	215/4883 (4,4)	0,129^a^	105 / 4883 (2,2)	0,028^a^	377 / 4883 (7,7)	0,228^a^
Tos crónica	5417 / 6238	86,8	237/5417 (4,4)	0,060^a^	122 / 5417 (2,3)	0,330^a^	411 / 5417 (7,6)	0,024^a^
Vómitos	5282 / 6247	84,6	244/5282 (4,6)	0,612^a^	120 / 5282 (2,3)	0,322^a^	399 / 5282 (7,6)	0,027^a^
Antecedentes familiares								
Equinococosis	6289 / 6548	96,0	272/6289 (4,3)	0,001^a^	147 / 6289 (2,3)	0,436^a^	503 / 6289 (8,0)	0,872^a^
Cisticercosis	6100 / 6526	93,5	274/6100 (4,5)	0,976^a^	133 / 6100 (2,2)	0,001^a^	505 / 6100 (8,3)	0,003^a^
Fascioliasis	6574 / 6658	98,7	287/6574 (4,4)	<0,001^a^	150 / 6574 (2,3)	0,711^a^	504 / 6574 (7,7)	<0,001^a^

a Prueba Chi Cuadrado de Pearson.

b Prueba exacta de Fisher.

## DISCUSIÓN

El Laboratorio de Referencia Nacional de Zoonosis Parasitaria del Instituto Nacional de Salud decide realizar una vigilancia basada en laboratorio para conocer la magnitud de la infección de fascioliasis, equinococosis quística, y cisticercosis de *T. solium* en 7811 muestras provenientes de 13 regiones del Perú. Los datos de dicha actividad han permitido identificar una seropositividad de 4,9% para equinococosis quística, de 7,9% para fascioliasis humana, y de 2,3% para cisticercosis de *T. solium*.

En cuanto a la seropositividad de equinococosis quística, se destaca que Cerro de Pasco (24,5%), Ayacucho (8%), Huancavelica (7,1%), Lima Provincias (6,1%), Junín (5,3%) y Cusco (4,3%) tuvieron las mayores frecuencias debido a que son consideradas zonas endémicas, tal como se evidencia en otros estudios realizados en los andes del Perú ^21^^-^^24^. Además, se encontraron casos de equinococosis quística en Huánuco (1,9%), Tacna (0,8%), Arequipa (3,7%), Apurímac (3,3%) y Puno (3,1%). Cabe mencionar que, en las regiones de Puno y Moquegua, se obtuvo una poca cantidad de muestras debido a que estas regiones no contaban con la logística necesaria para obtener una mayor cantidad de muestras en la vigilancia basada en laboratorio de las zoonosis parasitarias.

En el caso de fascioliasis humana, las regiones de Huánuco (8,6%), Ayacucho (6,2%) y Apurímac (5,9%) presentaron la mayor frecuencia de seropositividad, sin embargo, debemos resaltar también la presencia de casos en Cerro de Pasco (1,3%), Huancavelica (3,6%) y Tacna (1,5%) debido a que el presente estudio correspondería al primer reporte de casos de fascioliasis humana en estas regiones. Por lo tanto, es de suma importancia realizar otros estudios que permitan identificar zonas de riesgo para el control y prevención de esta zoonosis.

La frecuencia de fascioliasis humana fue mayor en Puno (40,6%), Cajamarca (20,0%), Arequipa (15,4%), Junín (5,5%) y Cusco (6,9%), que han sido identificadas como endémicas para esta etiología, tal como lo evidenció Marcos *et al.* en su reporte de casos humanos entre 1995 y 2005 ^25^, y en otros estudios que identificaron estas zonas como endémicas ^16^^,^^26^^,^^27^. Además de las regiones mencionadas, en Lima provincias, se encontró una alta seropositividad (13,1%). Este hallazgo concuerda con estudios previos, donde se ha reportado una prevalencia de 12,1% en Vichaycocha, Huancapón y Cajamarquilla de la sierra de Lima ^28^. Asimismo, la presencia de este parásito también ha sido reportado a partir de muestras coprológicas obtenidas en Huarochirí (16,7%) ^29^ y Oyón (1,12%) ^30^.

La seropositividad de cisticercosis de *T. solium* encontrada (2,3%) es menor a la prevalencia estimada en América Latina (4,08%) ^3^. Además, se encontraron casos de esta infección en regiones no reportadas anteriormente, como Huánuco (1,9%) y Huancavelica (0,5%). Se evidenció una seropositividad menor a los reportes preexistentes en algunas regiones, como Cerro de Pasco que antes reportó 4,2%, Cusco con 24,0% en Saylla, Junín con 7,0% en Huancayo, Apurímac con 12,0% en Andahuaylas, Tacna con 1,85% y Puno con 1,64% ^7^. Por otro lado, se encontraron regiones con una seropositividad mayor a lo antes reportado, como es Ayacucho que tenía reportes de 3,3% en Pampa Cangallo, Lima con seropositividad previa menor a 1%, y Arequipa que antes tenía 1,01% ^19^. Esta variación de resultados se puede deber a la cantidad de muestras obtenidas y las zonas de muestreo elegidas.

 Las características sociodemográficas que tenían una mayor seropositividad para estas zoonosis parasitarias fueron edad, y ocupación. Según los resultados obtenidos para la variable edad, existe una mayor frecuencia de anticuerpos tipo IgG contra equinococosis quística y cisticercosis de *T. solium* en personas adultas y adultos mayores, respectivamente, en comparación con la fascioliasis humana, que se encontró principalmente en adolescentes y adultos mayores. El incremento de la seropositividad de equinococosis quística y cisticercosis de *T. solium* con la edad encontrado en nuestro estudio es consecuente con lo hallado en otros estudios ^31^^,^^17^. Los grupos ocupacionales más afectados para estas zoonosis fueron las amas de casa y los agropecuarios, una mayor seropositividad de equinococosis quística en amas de casa ha sido reportado en otros estudios ^32^. Asimismo, el material de la vivienda, fuente de agua para consumo humano, consumo de agua no hervida y deposición en campo libre presentaron una diferencia en la seropositividad de fascioliasis humana, donde la pobreza y las condiciones sanitarias deficientes permiten la perpetuación de esta enfermedad, lo que ha sido encontrado en otros estudios ^8^^,^^26^^,^^33^.

Las costumbres y actividades de los pobladores de las zonas estudiadas que presentan una diferencia en la seropositividad de estas zoonosis fueron la crianza de animales como cerdos y ovejas en equinococosis quística; cabras, vacas y ovinos para fascioliasis humana, y la crianza de cerdos en campo libre para cisticercosis de *T. solium*. Estos animales actúan como hospederos que ayudan a que el ciclo biológico de los parásitos zoonóticos se complete. Al respecto, Ghatee *et al.*^34^, encontraron que la densidad poblacional del ganado ovino, vacuno y caprino influyen significativamente en la distribución de equinococosis quística. Además, se encontró relación entre una mayor frecuencia de cisticercosis de *T. solium* y el sacrificio de cerdos en el peridomicilio. Este hallazgo podría deberse a que la crianza de cerdo en condiciones insalubres y el consumo de su carne sin la inspección debida genera una mayor exposición a *T. solium*^35^. Por otro lado, los perros son utilizados para la crianza del ganado vacuno, ovino y caprino en el campo. Esto genera que los perros no desparasitados que consumen vísceras crudas, de animales sacrificados en el peridomicilio, sean una fuente de infección primaria de equinococosis quística para humanos y animales, lo que ha sido demostrado en el presente estudio debido a que se encontró una diferencia en la frecuencia de casos entre tener perros no desparasitados y el sacrificio de animales en el peridomicilio con la equinococosis quística.

Las zoonosis parasitarias se pueden adquirir por consumir agua y plantas contaminadas con huevos de *E. granulosus*, *T. solium*, o metacercarias de *F. hepatica*. Este hecho podría explicar nuestros resultados, los cuales, muestran que consumir verduras crudas en jugos, extractos y emolientes tuvo una diferencia en la seropositividad de equinococosis quística y fascioliasis humana. Este hallazgo ha sido determinado en estudios previos que reportaron resultados similares ^11^^,^^35^.

En cuanto a las variables clínico-epidemiológicas, se encontró una diferencia estadísticamente significativa en la frecuencia de casos entre la disminución de peso y dolor torácico con la seropositividad a equinococosis quística. Himsawi *et al.* encontraron que la disminución de peso es una característica asociada a la equinococosis quística ^20^. Además, otro estudio encontró una mayor frecuencia de dolor abdominal y tos crónica ^36^, tal como se observa en nuestro estudio. Los síntomas como dolor de cabeza, mareos y náuseas tuvieron mayor seropositividad de cisticerco de *T. solium* como lo demuestran estudios anteriores ^37^^,^^38^. Sin embargo, a pesar de que la cisticercosis está asociada con la epilepsia, en nuestro estudio no identificamos una diferencia en la frecuencia de casos. Este hallazgo podría atribuirse a que, en el presente estudio, solo se evaluaron datos centrados en la detección de anticuerpos circulantes tipo IgG, lo que no permitirá determinar una neurocisticercosis. Para fascioliasis, se encontró una diferencia significativa en la frecuencia de casos con la disminución de peso, esta diferencia ha sido encontrada también por Orfanos *et al.*^39^ en un estudio realizado en niños de tres provincias de Cajamarca. Por otro lado, el dolor abdominal y fiebre fueron síntomas frecuentes en la seropositividad a fascioliasis, lo que se condice con investigaciones anteriores ^40^^,^^41^.

Asimismo, se encontró que los participantes que tenían antecedentes familiares tuvieron una mayor frecuencia de las parasitosis estudiadas, lo que ha sido reportado previamente por Carmona *et al.*^42^. Este hallazgo podría deber a que la convivencia de las personas en un mismo ambiente y compartir los mismos alimentos y costumbres facilitaría la transmisión de estas zoonosis. Por ello, se recomienda tamizar a toda la familia una vez confirmado un caso de estas etiologías. Estos hallazgos demuestran la importancia de la detección temprana para realizar un tratamiento adecuado de estas zoonosis parasitarias para evitar complicaciones y de esta manera mejorar la salud pública.

Es importante indicar que, nuestro estudio presenta como principal limitación, la utilización de datos secundarios obtenidos previamente en actividades de vigilancia y cuyos datos no poseen una definición establecida en algún manual o guía para las fichas empleadas, sin embargo, la mayoría de los datos presentan claridad y reducida subjetividad. Así también, por la misma naturaleza del estudio de abordar datos secundarios no fue posible incorporar otras variables que pudieran ser de interés epidemiológico, como la variable «Ocupación» que fue recogida de forma abierta en la ficha, y fue categorizada por los autores de forma *ad hoc* según afinidad y similitud de las respuestas. Adicionalmente, los datos recolectados mediante las fichas epidemiológicas fueron empleadas para establecer, de forma exploratoria, una diferencia en la cantidad de casos identificados para cada una de las infecciones parasitarias. Otra limitación importante comprende a la representatividad de los datos analizados, dado que se tuvo una limitada cantidad de sujetos tamizados por región, así como por la selección no probabilística realizada. Estos aspectos sobre la obtención de los datos responden a dificultades logísticas, ya que los puntos de muestreo se encontraban en zonas rurales alejadas de la ciudad, donde era necesaria una gran capacidad logística. Debido a la baja representatividad por el diseño muestral y metodológico seguido durante el tamizaje (aspectos que afectan la validez externa de los datos), no fue posible establecer estimadores de prevalencia y/o seroprevalencia, sino únicamente de frecuencia de casos. Las limitaciones señaladas deben ser consideradas en la lectura crítica de este artículo. Sin embargo, a pesar de las limitaciones en del diseño muestral de este estudio, debemos resaltar que no se ha encontrado algún estudio previo con un tamaño de muestra amplio. Por este motivo, la información generada resulta de gran utilidad para conocer la distribución de estas infecciones. Asimismo, se debe considerar que en el Perú existe un subregistro de estas zoonosis parasitarias, debido a que son enfermedades desatendidas que se encuentran frecuentemente en zonas rurales en pobreza extrema, y que el Ministerio de Salud del Perú (MINSA) no tiene implementada la notificación obligatoria para la vigilancia, prevención y control de estas zoonosis parasitarias.

En conclusión, se ha podido obtener un mejor panorama en cuanto a la distribución y características de la afectación de estas zoonosis parasitarias en las 13 regiones del país, que se ha realizado mediante el análisis serológico de 7811 muestras obtenidas mediante actividades de vigilancia epidemiológica. Nuestros hallazgos podrían ser una herramienta útil para los tomadores de decisiones de los diversos sectores, tales como Salud, Agricultura y Medio Ambiente, para definir áreas estrategias y/o políticas con priorización o enfoque diferenciado para el control y prevención de estas zoonosis.
